# Genetic architecture of bone strength and composition in White Leghorn laying hens

**DOI:** 10.1186/s12711-026-01103-3

**Published:** 2026-11-03

**Authors:** Khrystyna Kurta, Peter W. Wilson, Björn Andersson, Matthias Schmutz, David Cavero, Tattiana Enrich-Essvein, Laura Crespo-Lopez, Alejandro B. Rodriguez-Navarro, Ian C. Dunn, Dirk-Jan de Koning, Martin Johnsson

**Affiliations:** 1 Swedish University of Agricultural Sciences, Uppsala, Sweden; 2 Roslin Institute, University of Edinburgh, Scotland, UK; 3 Lohmann Breeders, Cuxhaven, Germany; 4 H&N International, Cuxhaven, Germany; 5 Departamento de Mineralogia y Petrologia, Universidad de Granada, Granada, Spain

## Abstract

**Background:**

Skeletal issues such as fractures and poor bone quality are increasingly recognized for their negative impacts on animal welfare and the economic challenges they bring to the poultry industry. Previous pedigree-based analyses in White Leghorn laying hens have reported moderate heritabilities for cortical and medullary bone composition traits (ranging from 0.18 to 0.41) and strong genetic correlations (0.69 to 0.90) with tibia breaking strength and density. This study uses genome-wide association studies (GWAS) to identify genetic markers associated with bone strength and composition traits, aiming to advance our understanding of the genetic architecture of bone quality in White Leghorn laying hens.

**Results:**

We identified a set of markers on chromosome 20 (11.55–12.29 Mb) in strong LD with each other (*r*^*2*^ > 0.8), significantly associated with humeral stiffness, and overlapping with humeral strength and tibial cortical thickness. These markers were located within the previously identified QTL on chromosome 20 for bone biomechanical strength and body weight. This region harbors a *bone morphogenetic protein 7 *gene (*BMP7*) with a known role in skeletogenesis and growth in chicken. Genetic markers with the strongest suggestive association with organic and mineral contents, and total amount of carbonate in bone mineral MinCO3 1415 in tibia medullary bone were detected on chromosome 1 (173.5–173.6 Mb). This region contains the long non-coding RNA*LOC107052041 (LncFAM)*, which has previously been mapped within growth-related QTL and its polymorphisms have been associated with body weight and skeletal dimensions in chickens. Finally, more accurate heritabilities were estimated for thermogravimetry measurements of bone compositional traits as compared to infrared spectroscopy, with medullary organic and mineral contents being the most heritable (~0.19) and highly correlated with tibia density (0.7).

**Conclusions:**

This study identified genomic loci associated with variation in bone quality traits, providing candidates for functional validation and for future evaluation of their potential to improve the prediction of bone strength in laying hens.

**Supplementary information:**

The online version contains supplementary material available at https://doi.org/10.1186/s12711-026-01103-3.

## Background

Bone composition, structure, and strength are crucial for the health, productivity, and welfare of hens, as they experience problems with bone fracture, particularly of the keel, due to multiple factors, including the sustained calcium demands associated with egg production [1–3]. Keel bone has been described as especially vulnerable to fractures, with prevalence rates ranging from 3% at the start of lay to as high as 97% towards the end of the laying period [4–6]. Although hens can maintain high egg production during the peak laying period [7], the presence of a fresh keel bone fracture prompts hens to redirect resources toward healing, potentially impacting overall resilience [8]. Towards the end of the laying cycle, the combined physiological demands of egg production and fracture healing negatively impact productivity [7]. This indicates that hens face significant challenges in balancing the metabolic requirements of both processes, production, and fracture recovery [9]. Addressing these issues through genetic improvements in bone traits could help reduce the impact of fractures and improve overall flock welfare and productivity.

Earlier studies have demonstrated that genetics plays a role in bone strength, with heritability estimates in different genetic lines ranging from 0.19 to 0.59 [10–13]. Although the reported data show that the estimates highly differ between genetic lines [11]. In addition to bone strength, bone chemical composition also plays a crucial role in determining bone quality and structural integrity. Recent research has highlighted the genetic basis of bone chemical composition traits with heritability estimates of 0.18 - 0.41 in White Leghorn and Rhode Island Red [11]. This study identified significant genetic correlations (from 0.6 to 0.9) between medullary mineral content with tibia breaking strength or density. Further, genome-wide association studies in the Rhode Island Red breed identified genomic associations with cortical lipids. However, despite these advances, the genetic architecture underlying similar traits in White Leghorn breeds remains insufficiently explored, highlighting the need for further investigation through genomic studies.

In response to this knowledge gap, the current study aims to expand our understanding of the genetic factors influencing bone strength and compositional traits in White Leghorn laying hens using genomic data. Specifically, the objective of this study was to perform a genome-wide association study to detect genetic markers associated with these traits.

## Methods

### Study population

The study population comprised 987 White Leghorn laying hens from a pure breeding line maintained by Lohmann Breeders GmbH (Germany). All hens belonged to the same generation and were hatched during a four-week period in the same year. The population had a mixed paternal half- and full-sib family structure. A detailed description of population, housing system, and feeding is given in [11]. Body mass of the birds was recorded at 70 weeks and averaged at 1716.66 ± 127.68 g.

### Bone sampling

Birds were euthanized at 100 weeks of age, and bone samples were collected for further detailed bone measurements. In this study, we analyzed both the humerus and tibia, components of the appendicular skeleton, along with the keel (sternum), a component of the axial skeleton. Tibia bone quality was measured postmortem. We evaluated bone mechanical traits, density, morphometric, and composition traits. The studied bone traits were grouped into four categories: (i) bone mechanical traits, including breaking strength and stiffness; (ii) bone density traits; (iii) tibial morphometric traits; and (iv) bone composition traits measured by thermogravimetry and infrared spectroscopy.

### Bone mechanical traits

The mechanical properties of the tibia and humerus bones were assessed by breaking strength and stiffness. The detailed methodology of bone strength phenotype recording is presented in the previous study [11]. Briefly, bone breaking strength and stiffness were measured through a three-point bending test using a material testing machine (JJ Lloyd LRX50, Sussex, UK) described in [14].

### Bone density and morphometric parameters

Density of whole tibia, humeri, and keel bones was measured using Faxitron 43855D soft X ray apparatus fitted with an NTB EZ240 digital X-ray scanner (NTB GmbH, Germany). The exposure voltage was adjusted according to the age of the hen. Subsequently, the obtained X-ray images were scanned, and the bone was distinguished from the background. The mean radiographic density of the entire bone, pre-calibrated in millimeters of aluminum equivalent (mm AI equivalent), was then measured following the previously described methodology [11].

In addition to bone mechanical and density traits, we measured tibial morphometric parameters, including cortical diameter and thickness. The trait definitions and summary statistics for the studied bone mechanical properties, density, and tibial morphometric traits are presented in Table 1, while the trait phenotypic distributions are shown in Additional file 1, Figure S1.

**Table 1:** Trait definition, summary statistics, and heritability estimates for bone mechanical, density and morphometric traits

Trait	Description	N	Mean ±SD	h^2^ ±SE
**Bone mechanical traits**				
Tibia breaking strength	Tibiotarsus breaking strength (N)	891	216.95 ± 55.7	0.31 ± 0.05
Tibia stiffness	Tibiotarsus stiffness (N/m)	891	389534.55 ± 89499.9	0.23 ± 0.05
Humerus breaking strength	Humerus breaking strength (N) reflects its ability to withstand forces and resist fractures.	836	104.22 ± 25.3	0.27 ± 0.05
Humerus stiffness	Humerus stiffness (N/m) reflects the resistance of the bone to deformation under load.	840	214988.97 ± 58148	0.29 ± 0.05
Humerus bone score	Score for medullary bone content of humerus bone (score)	845	0.57 ± 0.9	0.16 ± 0.05
**Bone density traits**				
Tibia density	Tibiotarsus density (mm Al equivalent)	892	2.17 ± 0.2	0.33 ± 0.05
Humerus density	Humerus density (mm Al equivalent)	892	1.28 ± 0.3	0.10 ± 0.04
Keel RD	Whole keel radiographic density (mm Al equivalent)	899	0.81 ± 0.1	0.08 ± 0.04
**Bone morphometric traits**				
Tibia cortical diameter	Cortical mean diameter (mm)	903	6.83 ± 0.4	0.16 ± 0.05
Tibia cortical thickness	Cortical mean thickness (mm) generally contributes to higher bone strength and resistance to fractures.	910	0.49 ± 0.1	0.10 ± 0.04

*N, number of records; Mean ±SD, trait phenotypic mean and standard deviation; *h*^*2*^, heritability; SE, standard error. Heritability estimates are presented as *h*^*2*^ ± SE

### Bone chemical composition

The chemical composition of the bone-mid-shaft cortex and medullary bone was assessed using Fourier transform infrared spectroscopy (FTIR) and thermogravimetry (TGA), as detailed in [15]. In short, a 1-cm segment was cut from the mid-shaft of the tibia or humerus. Subsequently, cortical and medullary bone tissues were manually separated and then homogenized through grinding. The resulting cortical or medullary bone powders were analyzed in reflection mode using an FTIR spectrometer (model 6200, JASCO) equipped with an ATR unit (MIRacle Single Reflection ATR, PIKE Technologies). Infrared spectra were recorded at a resolution of 2-cm^−1^ over 100 scans. The compositional properties were determined based on the peak area of absorption bands associated with the bone’s chemical composition. In addition, the absolute water, organic matter, and carbonate contents were determined by thermogravimetry (TGA). The following procedure was used: about 25 mg of the powdered bone was introduced into a crucible and analyzed using a TGA system from METTLER-TOLEDO (mod. TGA/DSC1). A heating rate of 20 °C/min was used for registering the TGA curves. The recorded parameters, along with their detailed descriptions and summary statistics, are provided in Table 2. The trait phenotypic distributions are shown in Additional file 1, Figures S2 and S3.

**Table 2:** Trait definition, summary statistics, and genetic parameters for tibial cortical and medullary bone chemical composition traits

Trait	Description	N	Mean ±SD	h^2^ ±SE	Genetic correlation with Tibia density
**Tibial cortical bone: TGA**					
Cortical water	Water content (%)	903	10.64± 0.9	0.02 ± 0.03	0.24 ± 0.58
Cortical organic	Organic (collagen) matter weight (%).	897	24.84± 1.3	0.09 ± 0.04	−0.48±0.26
Cortical carbonate	Calcium carbonate content (CO3, %) which is determined as the weight loss in the 600–800 °C range (the remaining weight at 800 °C is phosphate (PO4%) in the mineral).	898	3.64 ± 0.6	0.03 ± 0.03	−0.06±0.49
Cortical mineral	Minerals weight (%); calculated as the sum of carbonate (%) and phosphate (%).	898	64.49± 1.4	0.09 ± 0.03	0.37 ± 0.21
**Tibial cortical bone: FTIR**					
Cortical PO4/Amide I	Relative amount of mineral to organic matrix (Amide I) in tibia bone which indicates the degree of mineralization in bone.	893	3.5 ± 0.8	0.01 ± 0.001	−0.98±0.59
Cortical MinCO3 870	The amount of carbonate substituted in the mineral as the ratio of CO3 (870 cm^−1^) to the main phosphate (900 to 1200 cm^−1^) band area. This measurement is related to bone mineral maturation.	897	0.07 ± 0.0	0.001 ± 0.0	0.95 ± 0.8
Cortical MinCO3 1415	The total amount of carbonate in bone mineral; determined as the ratio of the main carbonate band (1415 cm^−1^) to the main phosphate (900 to 1200 cm^−1^) band area ratio. This is an indicator of bone mineral maturation and bone remodeling rate (decreases with bone mineral maturation and bone tissue age [16].	899	0.25 ± 0.0	0.03 ± 0.03	0.65 ± 0.92
Cortical CO3/Amide I	Carbonate (CO3, at main peak 1415 cm^−1^) relative to organic matter (Amide I, at main peak 1640 cm^−1^); it refers to the degree of bone mineralization process [15].	894	0.85 ± 0.1	0.01 ± 0.001	−0.98 ±0.66
Cortical CO3 1450/1415	Ratio of secondary carbonate and organic matter peak (1450 cm^−1^) to the main carbonate peak (1415 cm^−1^). Associated with the substitution of carbonate within the bone mineral.	901	0.81 ± 0.1	0.03 ± 0.03	0.23 ± 0.5
Cortical CO3 870/1415	Ratio of secondary carbonate and organic matter peak (870 cm^−1^) to the main carbonate peak (1415 cm^−1^). This ratio is used to assess the relative amount of secondary carbonate and organic matter compared to the primary carbonate.	899	0.28 ± 0.1	0.01 ± 0.00	−0.99 ±0.0
Cortical crystallinity index	Crystallinity index of the bone determined through the ratio between the peak areas at 1030 cm^−1^ to 1020 cm^−1^. It provides information about the degree of crystallinity of the bone’s mineral phase.	906	0.52 ± 0.1	0.04 ± 0.03	0.30 ± 0.64
Cortical collagen maturity	Mature relative to immature collagen cross-links; mature and immature collagen measured as the FTIR area at main peak 1660 cm^−1^ and 1690 cm^−1^, respectively; this measurement is used as an indicator of the collagen maturity.	893	3.78 ± 0.9	0.02 ± 0.02	−0.99 ±0.09
Cortical lipids	Carbonyl group from the lipid; measured as the FTIR area at main peak 1710 cm^−1^.	896	0.02 ± 0.0	0.05 ± 0.03	−0.01 ±0.38
Cortical sulfates	Sulfate content. If this value is high, it changes the bone properties.	892	0.17 ± 0.0	0.05 ± 0.03	0.09 ± 0.39
Cortical Amide II/Amide I	Ratio of Amide II relative to organic matter (Amide I) which can suggest alterations in the collagen secondary structure, reflecting bone integrity.	903	0.86 ± 0.1	0.06 ± 0.04	−0.08 ±0.36
**Tibial medullary bone: TGA**					
Medullary water	Medullary water weight (%)	928	9.94 ± 1.7	0.01 ± 0.02	0.31 ± 1.15
Medullary organic	Medullary (collagen) matter weight (%)	926	51.11 ± 9	0.19 ± 0.07	−0.7 ±0.08
Medullary carbonate	Medullary carbonate weight (CO3, %)	911	5.31 ± 6.8	0.01 ± 0.01	−0.27 ±0.48
Medullary mineral	Minerals weight (%); calculated as the sum of carbonate (%) and phosphate (%).	923	38.73 ± 8.4	0.18 ± 0.07	0.72 ± 0.08
**Tibial medullary bone: FTIR**					
Medullary PO4/Amide I	As in cortical	924	0.69 ± 0.3	0.07 ± 0.05	−0.21 ±0.46
Medullary MinCO3 870	As in cortical	931	0.01 ± 0	0.03 ± 0.02	−0.14 ±0.62
Medullary MinCO3 1415	As in cortical	918	0.51 ± 0.2	0.02 ± 0.02	0.87 ± 1.24
Medullary CO3/Amide I	As in cortical	913	0.33 ± 0.1	0.01 ± 0.0	0.87 ± 1.44
Medullary CO3 1450/1415	As in cortical	914	0.96 ± 0.1	0.03 ± 0.03	−0.05 ±0.39
Medullary CO3 870/1451	As in cortical	921	0.03 ± 0	0.04 ± 0.05	−0.21 ±0.49
Medullary crystallinity index	As in cortical	932	0.58 ± 0.4	0.002 ± 0.01	0.99 ± 0.61
Medullary collagen maturity	As in cortical	923	3.45 ± 0.4	0.05 ± 0.06	0.28 ± 0.37
Medullary lipids	As in cortical	924	11.45± 25.4	0.02 ± 0.02	−0.27±0.63
Medullary sulfates	Sulfates content	921	0.12 ± 0	0.12 ± 0.07	−0.26 ±0.34
Medullary Amide II/Amide I	As in cortical	924	0.84 ± 0.1	0.06 ± 0.06	0.22 ± 0.55

*N*, number of records; Mean ±SD, trait phenotypic mean and standard deviation; *h*^*2*^, heritability; SE, standard error. Heritability estimates are presented as *h*^*2*^ ± SE

TGA, thermogravimetric analysis; FTIR, fourier-transform infrared spectroscopy

Tibial density was selected as the reference trait for assessing genetic associations between bone composition and whole-bone mechanical and density traits, as it had the highest heritability among these traits

### Genotyping and quality control

All hens were genotyped with a 57,636 Illumina Infinium SNP array at the SNP&SEQ Technology Platform (Uppsala University, Sweden). Sequences flanking the SNPs were aligned against the GRCg6a chicken reference genome (GenBank assembly accession: GCA 000002315.6) to find their coordinates on this assembly. Monomorphic SNPs with low call rates (<90%) and birds with low call rates (<95%) were removed, following [10]. In total, 17,633 SNPs and 987 birds passed all criteria and were used for the downstream analysis. All associations with MAF ≥ 0.05 were retained for the biological interpretation.

### Statistical analyses

#### Phenotypic data

Before all subsequent analyses, including genome-wide association study and estimation of phenotypic and genetic correlations, phenotypic data were filtered by removing outliers based on ± 3 standard deviations from the mean threshold using base packages in R software [17]. All traits were standardized in R using the following Eq. (1):

1 $${x_{new}} = {{{x_i} - \mu } \over \sigma }$$

where *x*_*i*_ is the phenotypic record of animal *i*, μ is the mean value, and σ is the phenotypic standard deviation.

Phenotypic correlations and the respective significance levels were estimated using the cor.test() function in R [17].

We performed a multiple regression analysis to identify potential covariates for inclusion in the genetic analyses. The hatch group was significantly associated (*p* < 0.05) with several traits used in this study. For example, it was associated with lower tibial density (*β*: −3.5), tibial breaking strength (*β*: −0.013), humeral stiffness (*β*: −3901.2), humeral breaking strength (*β*: −2.6), and tibial cortical diameter (*β*: −0.06), but with a higher tibial cortical mineral content (*β*: 0.09). Hatch group was therefore included as a categorical fixed effect, and it comprised four levels: group 1 (*n* = 234), group 2 (*n* = 238), group 3 (*n* = 239), and group 4 (*n* = 245). Body mass was included as a covariate in the analyses of bone strength and density traits to account for its influence on skeletal loading and mechanical strength. Bone compositional traits were analyzed without adjustment for body mass because our objective was to estimate their overall relationships on the observed scale. This approach was consistent with [11].

#### Genome-wide association study

A genome-wide association study was performed using the “–mlma”, “–grm”, and “–reml” options implemented in GCTA software v.1.92.3 [18]. This approach uses a linear mixed model with a correction for animal relatedness based on the genomic relationship matrix. The model jointly fits the fixed effect of the candidate SNP (to be tested for the association) and the random polygenic effect of other SNPs, including the candidate variant. As we determined before, block significantly affected the traits of interest and therefore was used as a fixed effect, while in the case of bone strength traits, body weight was additionally included as a covariate in the model Eq. (2):

2 $${\bf{y}} = {\bf{\it{\mu }}} + {\bf{Xb}} + {\bf{Z\alpha }} + {\bf{g}} {\bf{snp}} + {\bf{e}}$$

where ${\bf{y}}$ is the standardized trait measurement; ${\bf{\it{\mu }}}$ is the population mean of the variable of interest; ${\bf{X}}$ is a design matrix that relates **y** measurements to the vector ${\bf{b}}$ of confounding fixed effect; where **Z** is the incidence matrix linking the recorded animals with their genotypes, **α** is a vector of allele substitution effect for the candidate SNP, ${\bf{g}}$ is a vector of total SNP effects with **g***~N(0,*${\bf{\it{G}}}\sigma _{\bf{g}}^2$), where **G** stands for the SNP genetic relationship matrix between individuals and $\sigma _{\bf{g}}^2$ is variance explained by all the SNPs; **snp** is a vector of the SNP genotypes coded as 0, 1 and 2, respectively for common homozygous and heterozygous alleles, and rare homozygous alleles; ${\bf{e}}$ is a vector of random residual effects with **e***~N(0,*${\bf{\it{I}}}\sigma _{\bf{e}}^2$) where **I** is the identity matrix and ${\sigma _e}$ the residual variance. The genome-wide significance threshold was calculated using a Bonferroni correction using Eq. (3):

3 $$bonf = {{0.05} \over N},$$

where N is the number of SNPs that passed the quality control. A nominal threshold of 10^−3^ was considered as suggestive.

Manhattan plots and QQ plots were created using the ggplot2 package [19] implemented in R software [17].

#### Linkage disequilibrium

Linkage disequilibrium analysis was computed between each significant SNP (top SNP with *p* < 2.8 × 10^−6^) and the other SNPs located 1 Mb upstream and downstream of the top significant (lead) SNP. The LD level was estimated using the Plink v1.09 software [20, 21] with the following parameters: –ld-window-kb 1000, –ld-window 99,999, and –ld-window-r2 set to zero to generate the output file with all SNP pairs within 1Mb reported.

#### Candidate genes and identification of overlapped QTLs

Genomic regions with overlapped significant and suggestive SNP associations (*p* < 1 × 10^−3^) with bone mechanical, density, and compositional traits were tested for proximity to a gene located within 100 kb upstream or downstream of each SNP. For a more focused positional candidate-gene analysis, we retained all annotated genes located within 100 kb upstream or downstream of SNPs reaching the significance threshold (*p* < 1 × 10^−5^). The available literature for these candidate genes was reviewed to summarize the current knowledge of the functions and previously reported trait associations of every gene. In addition, we used the NCBI gene annotation file for the GRCg6a chicken reference genome (GenBank assembly accession: GCA 000002315.6, Annotation Release 104). Information on the gene function of the detected genes was obtained from the literature. Subsequently, we run functional annotation and enrichment analysis of gene lists using DAVID [22].

Phenotypic correlations and the respective significance levels were estimated using the cor.test() function in R [17].

We performed a multiple regression analysis to identify potential covariates for inclusion in the genetic analyses. Hatch group was significantly associated (*p* < 0.05) with several traits used in this study. For example, it was associated with lower tibial density (*β*: −3.5), tibial breaking strength (*β*: −0.013), humeral stiffness (*β*: −3901.2), humeral breaking strength (*β*: −2.6), and tibial cortical diameter (*β*: −0.06), but with a higher tibial cortical mineral content (*β*: 0.09). Hatch group was therefore included as a categorical fixed effect, and it comprised four levels: group 1 (*n* = 234), group 2 (*n* = 238), group 3 (*n* = 239), and group 4 (*n* = 245). Body mass was included as a covariate in the analyses of bone strength and density traits to account for its influence on skeletal loading and mechanical strength. Bone compositional traits were analyzed without adjustment for body mass because our objective was to estimate their overall relationships on the observed scale. This approach was consistent with [11].

#### Genetic correlation and heritability

Genetic parameters were estimated by a bivariate model with AIREMLF90 [23] using the following Eq. (4):

4 $$\left[ {\matrix{ {{{\bf{\it{y}}}_1}} \cr {{{\bf{\it{y}}}_2}} \cr } } \right] = \left[ {\matrix{ {1{{\bf{\it{\mu }}}_1}} \cr {1{{\bf{\it{\mu }}}_2}} \cr } } \right] + \left[ {\matrix{ {{{\bf{\it{X}}}_1}} & 0 \cr 0 & {{{\bf{\it{X}}}_2}} \cr } } \right]\left[ {\matrix{ {{{\bf{\it{b}}}_1}} \cr {{{\bf{\it{b}}}_2}} \cr } } \right] + \left[ {\matrix{ {{{\bf{\it{Z}}}_1}} & 0 \cr 0 & {{{\bf{\it{Z}}}_2}} \cr } } \right]\left[ {\matrix{ {{{\bf{\it{u}}}_1}} \cr {{{\bf{\it{u}}}_2}} \cr } } \right] + \left[ {\matrix{ {{{\bf{\it{g}}}_1}} \cr {{{\bf{\it{g}}}_2}} \cr } } \right] + \left[ {\matrix{ {{{\bf{\it{e}}}_1}} \cr {{{\bf{\it{e}}}_2}} \cr } } \right],$$

where ${{\bf{\it{y}}}_1}{\bf{\it{ }}}$ and ${{\bf{\it{y}}}_2}$ are the vectors of the corresponding two phenotypic traits under study; ${\mu _1}$ and ${\mu _2}$ are their respective means; ${{\bf{\it{b}}}_1}$ and ${{\bf{\it{b}}}_2}$ are vectors of fixed effects with ${{\bf{\it{X}}}_1}$ and ${{\bf{\it{X}}}_2}$ their corresponding design matrices; ${{\bf{\it{u}}}_1}$**and**${{\bf{\it{u}}}_2}$*~N(0*, **G**_0_ ⨂ **I**), are vectors of random genetic effects (breeding values) for the traits, with ${{\bf{\it{Z}}}_1}$ and ${{\bf{\it{Z}}}_2}$ their respective design matrices; and ${{\bf{\it{e}}}_1}$ and ${{\bf{\it{e}}}_2}$*~N(0*, **R**_0_ ⨂ **I**) are vectors of residual errors. **G**_0_ and **R**_0_ are the 2 × 2 variance-covariance matrices for random effects and residuals, while ⨂ denotes the Kronecker product. **I** is the identity matrix.

The genomic relationship matrix was computed as shown in Eq. (5) [24]:

5 $${{{\bf{\it{Z}}}{\rm{ }}{{\bf{\it{Z}}}^{\rm{\prime}}}} \over {2{\rm{ }}\mathop \sum \nolimits {{\bf{\it{p}}}_{\bf{\it{i}}}}(1 - {{\bf{\it{p}}}_{\bf{\it{i}}}})}}$$

where **Z** is a matrix of centered genotypes and ${{\rm{p}}_{\bf{\it{i}}}}$ the corresponding MAF for each SNP.

Heritability estimates were obtained for the recorded traits using the following Eq. (6):

6 $${h^2} = {{\sigma _g^2} \over {\sigma _g^2 + \sigma _e^2}}$$

The genetic correlations among traits were calculated using AIREMLF90 with the following Eq. (7):

7 $${r_{i, j}} = {{{\sigma _{gi, gj}}} \over {\sqrt {\sigma _{gi}^2 \times \sigma _{gj}^2} }}$$

where ${\sigma _{gi, gj}}$ is the genetic covariance variance between the two traits, and $\sigma _{gi}^2$ and $\sigma _{gj}^2$ the corresponding additive genetic variances.

## Results

### Genome-wide association study

**Fig. 1:**
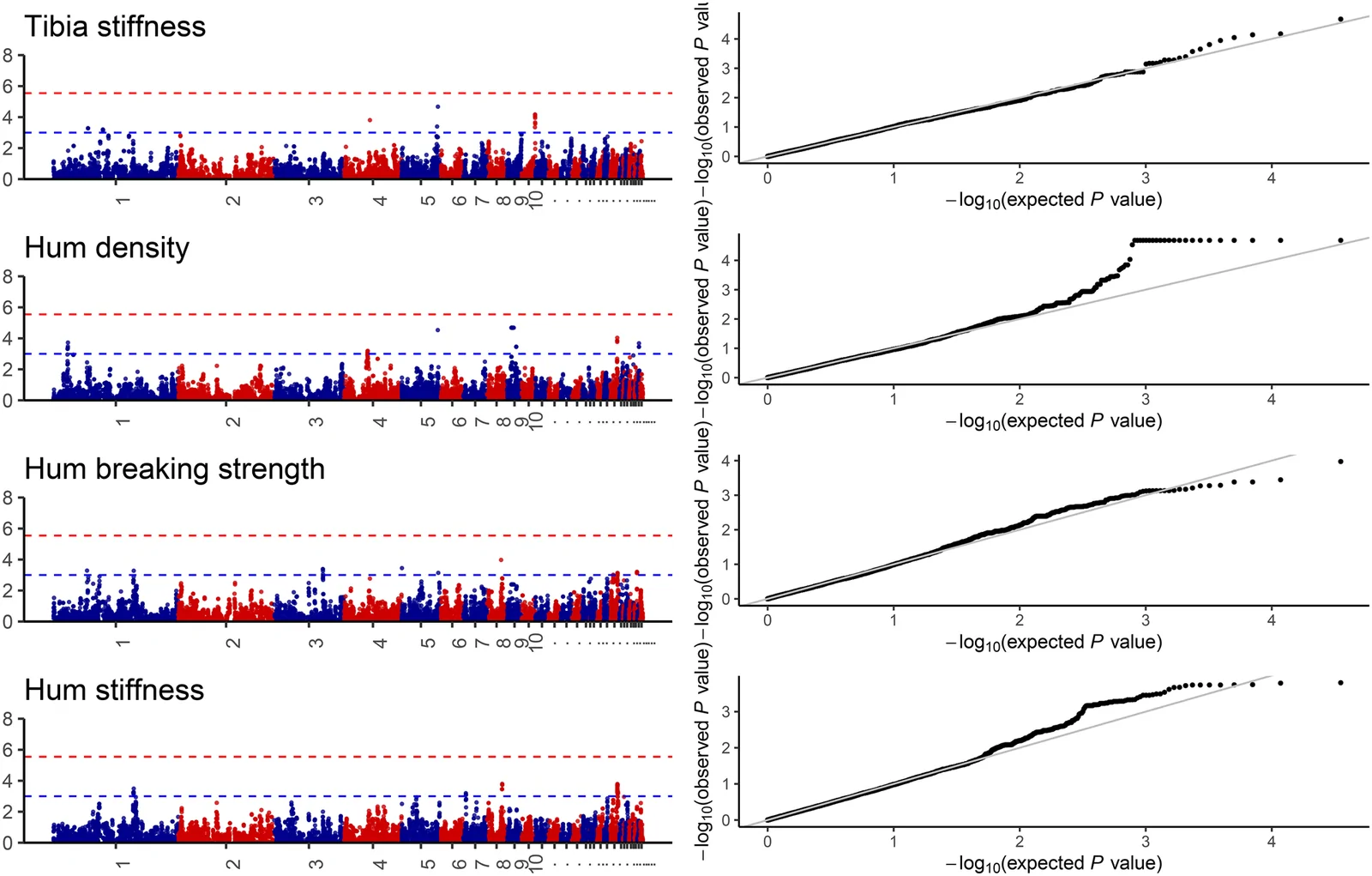
GWAS results for traits showing the strongest association signals: tibial stiffness, humeral density, humeral breaking strength, and humeral stiffness. Manhattan plot (left) showing the −log10(*p*) for each SNP, and QQ plot (right), showing the observed versus expected −log10(*p*). The red line indicates the Bonferroni significance threshold of *p* = 2.84 × 10^−6^, and the blue line indicates the suggestive significance threshold of *p* = 1 × 10^−3^. SNPs exceeding the Bonferroni significance threshold are highlighted in green

Our results revealed 13 SNPs below the significance threshold (*p* < 2.84 × 10^−6^) on chromosome 20: 11.59–11.78 Mb (MAF 0.4–0.5) associated with humerus stiffness, a single SNP (58,281,780 bp; MAF 0.2) associated with humerus density (chromosome 5), and three SNPs (55,553,280, 55,598,304, 55,662,347 bp; MAF 0.007) on chromosome 1 associated with tibia stiffness (Fig. 1). No SNP reached the specified significance threshold for the other traits analyzed.

We next identified suggestive associations (*p* < 1 × 10^−3^) for the studied bone mechanical, density, and compositional traits. In particular, SNPs above the suggestive threshold associated with these mechanical and density phenotypes were detected across 20 chromosomes. Notably, chromosome 5 contained suggestive SNPs associated with six of the ten phenotypes. Suggestive associations were detected across 10 chromosomes for the tibial traits, 17 chromosomes for the humeral traits, and five chromosomes for keel radiographic density (keel RD), whereas cortical thickness and diameter showed suggestive associations on nine chromosomes (Additional file 1, Figure S4).

Among bone chemical composition parameters, cortical TGA traits showed suggestive associations on six chromosomes, while for FTIR measured traits, we revealed SNPs below the suggestive threshold on 19 chromosomes (Additional file 1, Figure S5). In the medulla, TGA traits had suggestive associations on eight chromosomes. Medullary FTIR parameters were found to have a suggestive association across 15 chromosomes (Additional file 1, Figure S6).

### Overlap of genome-wide association results

SNPs associated with more than one trait were identified (Additional file 2, Table S1). Amongst the bone mechanical, density, and morphometric traits, the highest number of overlapping SNPs (34 SNPs) was found within a large QTL region on Chr 20 (11.55–12.29 Mb) for humerus breaking strength and stiffness and tibial cortical thickness. A cluster of five overlapping SNPs within the QTL region on Chr 3 (79.41–79.57 Mb) and four SNPs on Chr 22 (0.74–0.93 Mb) were detected between tibial traits (tibia breaking strength, stiffness, and density). These SNPs exhibited consistent positive or negative effects across traits, which aligns with the strong genetic correlation among them (Additional file 1, Figure S7b; Additional file 2, Table S2). Within a smaller QTL region on Chr 1 (55.55–55.66 Mb) and Chr 10 (21.04–21.20 Mb), three overlapping suggestive SNPs were found across tibia traits density and stiffness.

Among bone chemical composition traits in cortex, four SNPs on Chr 3 (85.34–85.53 Mb), six SNPs on Chr19 (0.13–0.51 Mb), and three SNPs on Chr 28 (2.06 - 2.09 Mb) showed suggestive associations with multiple FTIR traits, such as CO3 1415/Amide I and PO4/Amide I with SNP effects in the same direction. Another cluster of six SNPs on Chr 10: 18.53–18.68 Mb was associated with both CO3 870/1415 and MinCO3 870, both showing positive effects. In medulla, seven overlapping SNPs on Chr 1: 173.53–173.75 Mb displayed positive effects on medullary organic content (TGA) while showing negative effects on mineral content (TGA) and carbonate in bone mineral (MinCO3 1415, FTIR). Finally, several overlapping SNPs were identified between bone strength and compositional traits across multiple chromosomes. For example, on Chr 17: 0.96–0.99 Mb, three SNPs exhibited positive effects on humerus bone score but negative effects on cortical water (TGA). A complete list of SNPs associated with multiple traits, their respective effects, and *p* values is provided in Additional file 2, Table S1.

### Linkage disequilibrium

To further investigate linkage disequilibrium, we analyzed QTL regions showing the strongest association signals and the greatest overlap of associated SNPs across multiple traits. These include QTLs on Chr 1: 55.55–79.83 Mb (post-mortem tibial traits), Chr 20: 11.55–12.14 Mb (humeral bone strength traits), and Chr 1: 173.5–173.6 Mb (medullary organic, mineral content, and carbonate in bone mineral).

On Chr 1 (55.55–79.83 Mb), three SNPs significantly associated with tibia stiffness, and also associated with tibia breaking strength and density, were in complete LD with one another (*r*^*2*^ = 1.00; Additional file 1, Figure S8). Despite this, these SNPs had a very low minor allele frequency (MAF = 0.007), they were physically distant from other SNPs (>70 kb), and were not supported by neighboring SNPs in linkage disequilibrium. Therefore, these associations were considered spurious and were excluded from further biological interpretation.

**Fig. 2:**
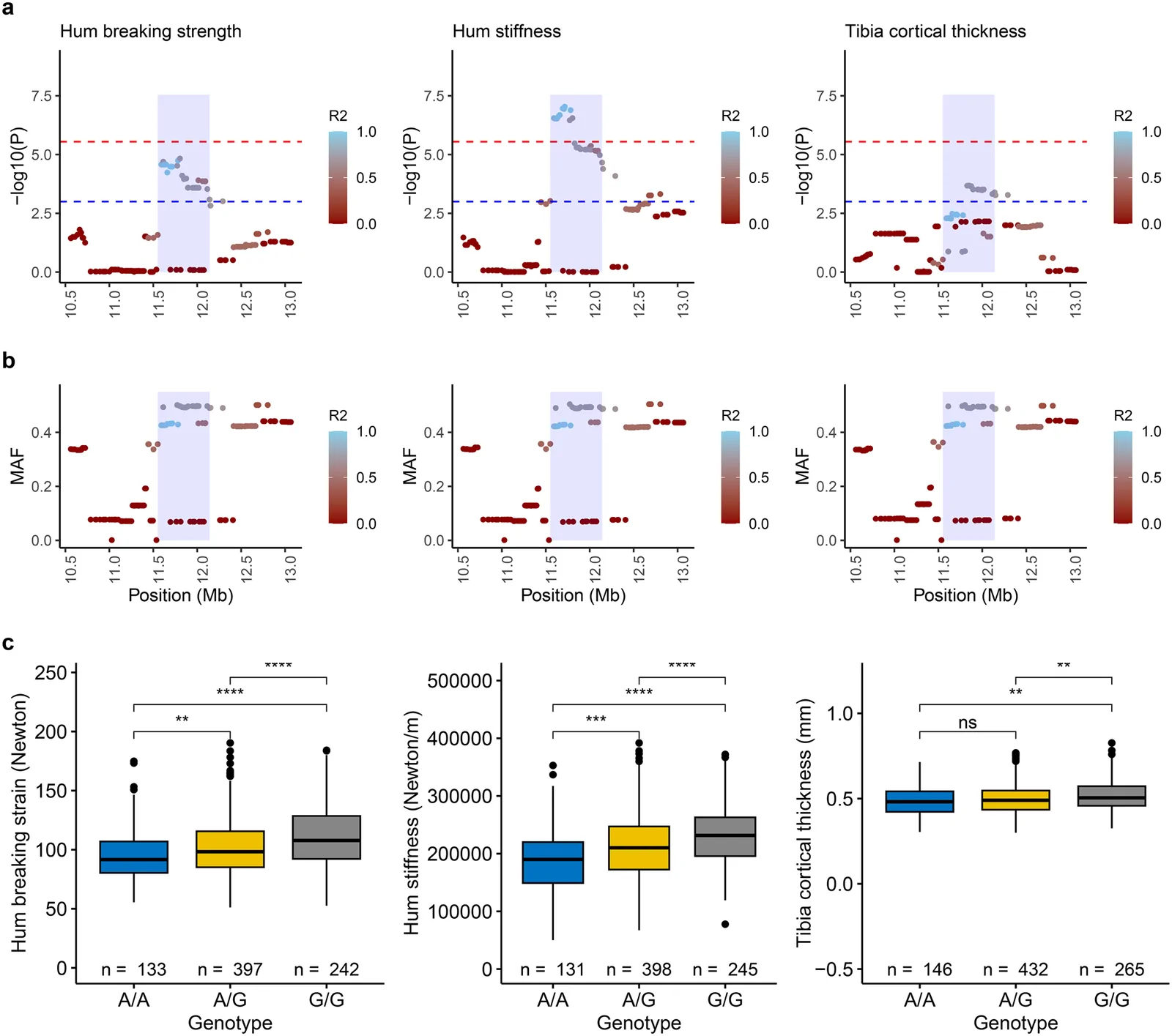
Regional association plots showing overlapping signals for humeral breaking strength, humeral stiffness, and tibial cortical thickness within the chromosome 20 QTL region (11.55–12.14 Mb). (**a**) LD plots showing the local LD structure around the most significant SNPs in the QTL region, including 1Mb upstream and downstream. Each dot represents an SNP and is colored according to its LD with the top SNP. The shaded rectangle highlights the QTL region. The red and blue lines indicate the Bonferroni significance (*p* = 2.84 × 10^−6^) and suggestive association (*p* = 1 × 10^−3^) thresholds. (**b**) Minor allele frequencies of SNPs across the displayed chromosome 20 region. (**c**) Boxplot showing the effect of the most significant SNP (Chr 20: 11,715,985 bp) on humerus bone breaking strength, stiffness, and tibia cortical thickness. Wilcoxon rank test *P*s: ** *p* < 0.01, ***p < 0.001, ****p < 0.0001

On Chr 20: 11.55–12.14 Mb, the top significant SNP (11,715,985 bp), associated with humerus breaking strength, was in strong LD (*r*^*2*^ > 0.8) with 13 nearby SNPs but showed lower LD with more distant SNPs (Fig. 2a). MAF distributions in the region were moderate (0.4–0.5), indicating these SNPs are common in the population (Fig. 2b). Notably, individuals with G/G genotype exhibited higher humeri breaking strength (6–16%), higher stiffness (11–21%), and higher cortical thickness (4–9%) compared to A/G or A/A genotype (Fig. 2c).

**Fig. 3:**
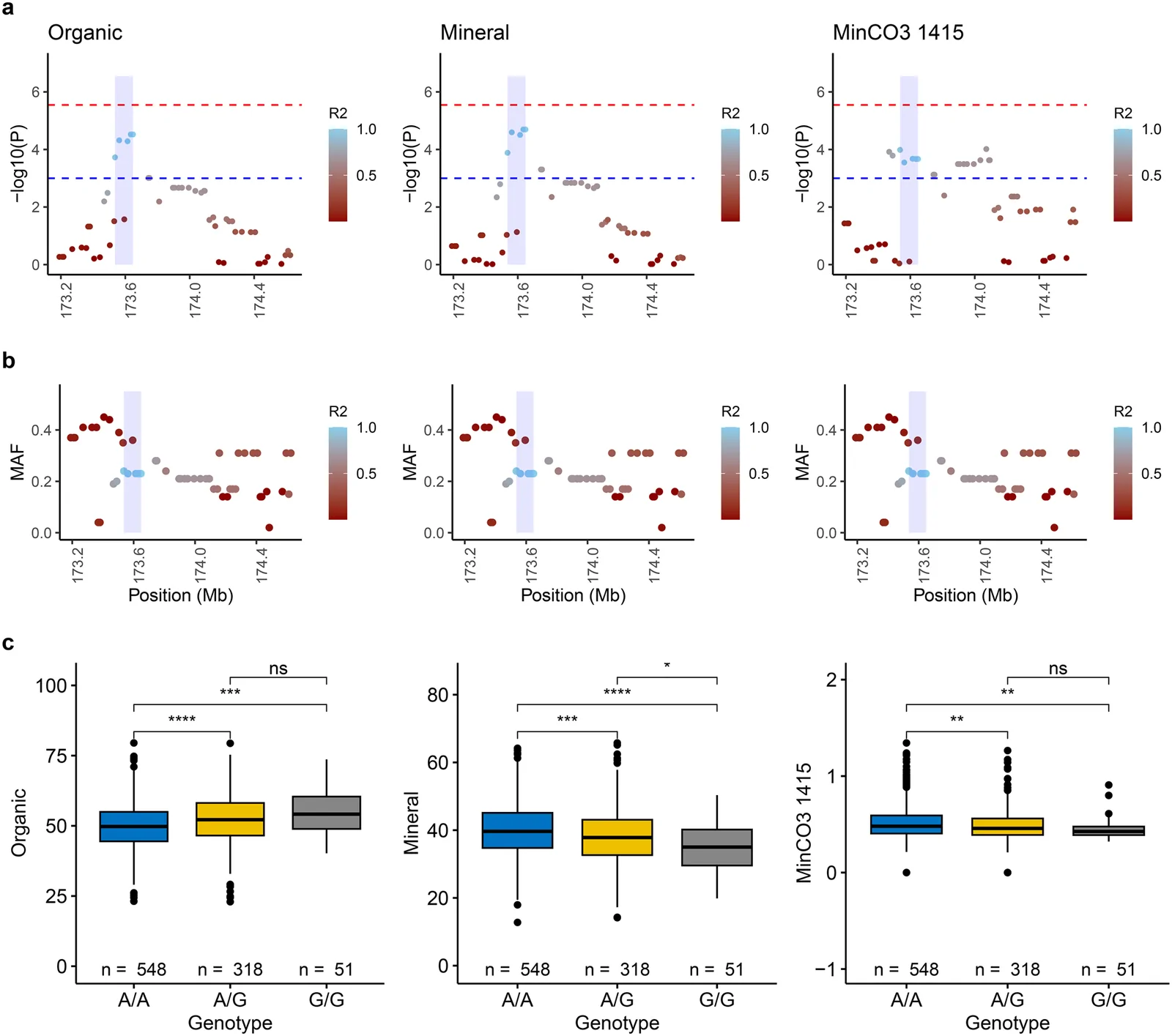
Regional association plots showing overlapping signals for medullary organic, mineral, and MinCO3 1415 contents within the chromosome 1 (173.53–173.75 Mb). (**a**) LD plots showing the local LD structure around the most significant SNPs in the QTL regions on chromosome 1, 1Mb upstream and downstream. Each dot represents an SNP marker, colored in accordance with its LD from the top SNP. The shaded rectangle highlights the region of interest at Chr 1: 173.53–173.75 Mb. The red and blue lines indicate the Bonferroni significance (*p* = 2.84 × 10^−6^) and suggestive association (*p* = 1 × 10^−3^) thresholds. (**b**) The minor allele frequencies in the chromosome region are depicted under the x-axis. (**c**) Boxplot showing the effect of one of the most significant SNPs (173,647,596 bp) on tibia medullary bone composition traits. Wilcoxon rank test *P*s: ** *p* < 0.01, ***p < 0.001, ****p < 0.0001

Seven overlapping suggestive SNPs on Chr 1: 173.5–173.6 Mb, associated with medullary organic content, mineral content and carbonate in bone mineral, were in high LD (*r*^*2*^ = 0.97–1.00) with the top SNP (173,634,193 bp) but showed lower LD (*r*^*2*^ = 0.11–0.78) with the surrounding SNPs (Fig. 3a). These SNPs were relatively common in the population (MAF = 0.23; Fig. 3b). Genotype analysis at Chr 1:173,634,193 bp revealed that the individuals with the A/A genotype had 6–13% higher TGA mineral content and 8–16% FTIR mineral carbonate content, but 5–10% lower organic content compared A/G and G/G genotypes (Fig. 3c).

### Gene annotation and enrichment analysis

Candidate regions with the suggestive associations (*p* < 1 × 10^−3^) and overlapping signals identified through GWAS (Additional file 2, Table S1) were further analyzed to identify positional genes. Gene annotation resulted in 255 unique genes 100 kb up- and downstream from the candidate markers (Additional file 2, Table S3). Although a high number of genes was identified, only 69 genes were reported by DAVID tool to be involved in eight significant processes (GO terms; Table 3).

**Table 3:** Gene set enrichment test involving the genes within 100 kb of the SNPs with *p* < 1 × 10^−3^

GO term	Number of genes	P
Arachidonic acid metabolism	6	0.001
Ether lipid metabolism	5	0.006
Alpha-Linolenic acid metabolism	4	0.007
Linoleic acid metabolism	4	0.011
Vascular smooth muscle contraction	7	0.013
Glycerophospholipid metabolism	6	0.018
Protein processing in endoplasmic reticulum	7	0.034
Metabolic pathways	30	0.061

**Fig. 4:**
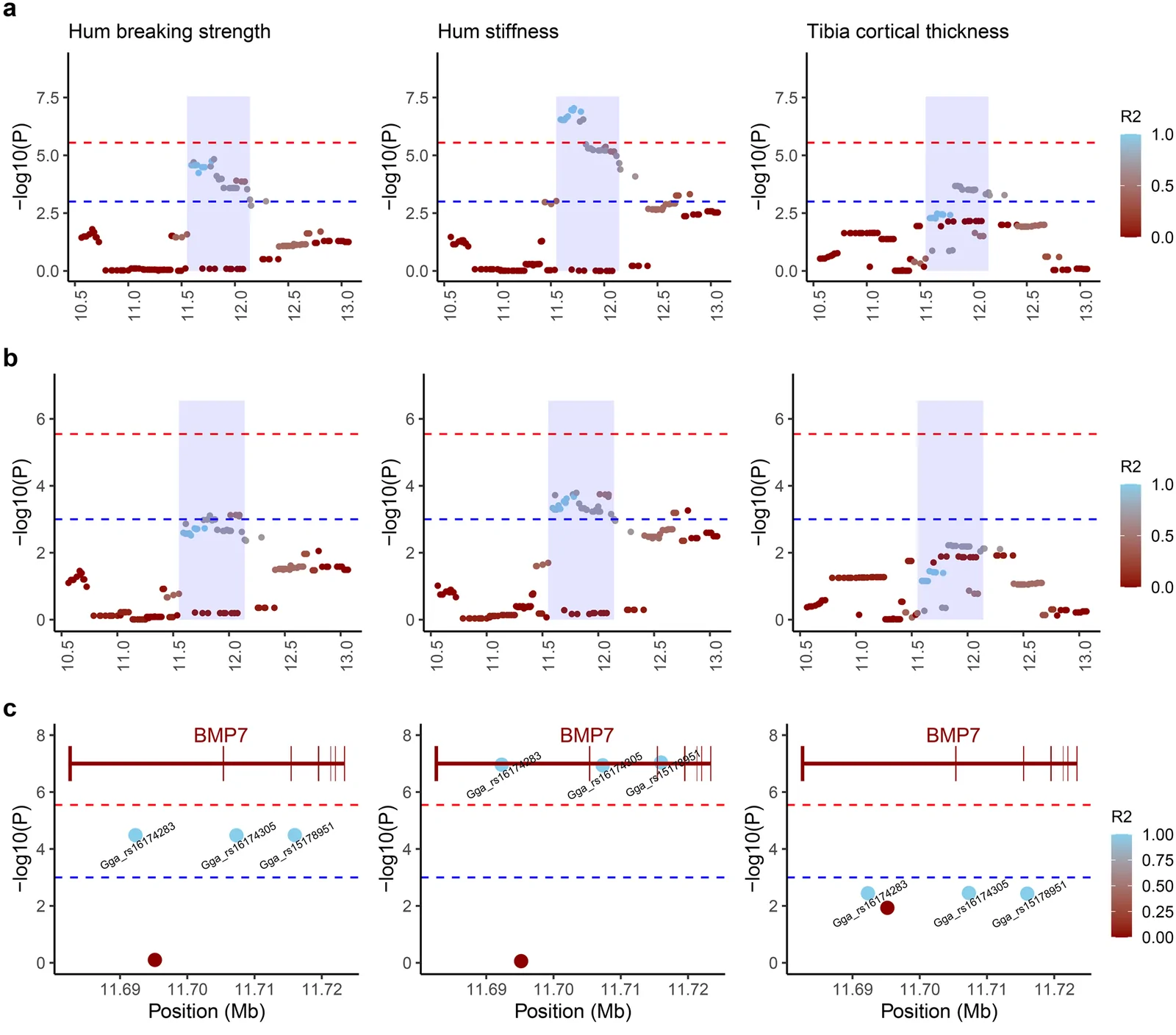
Local linkage disequilibrium (LD) structure around the most significant SNPs within the chromosome 20 QTL region associated with humeral breaking strength, humeral stiffness, and tibial cortical thickness. Plot (**a**) shows results without adjusting for body weight, while plot (**b**) presents the results adjusted for body weight. (**c**) Zoom-in profile of the GWAS results surrounding the *BMP7* gene position at chromosome 20: 11.68 - 11.72 Mb with the vertical rectangles indicating the coding sequence regions of the gene. Each dot represents a SNP marker, colored in accordance with their LD from the top SNP. The shaded rectangular highlights the region of interest at chromosome 20: 11.55–12.14 Mb. The red and blue lines indicate the Bonferroni significance (*p* = 2.84 × 10^−6^) and suggestive association (*p* = 1 × 10^−3^) thresholds

A more focused positional candidate-gene analysis identified 25 genes within the same ±100-kb window around all significant SNPs reaching *p* < 1 × 10^−5^. The latest knowledge on the gene functions is presented in Additional file 2, Table S4. Significant SNPs within Chr 20:11.55–12.14 Mb region associated with humerus bone stiffness and overlapped with suggestive SNPs for humeri bone breaking strength, and tibia cortical thickness were located within 15 genes, five out of which were reported to be involved in broad biological processes, but not explicitly in bone biology; nine genes were uncharacterized; and one gene was the bone morphogenetic protein 7 (*BMP7*; Fig. 4c; Additional file 2, Table S4) with a known role in various biological processes, including the process of skeletogenesis [25, 26] and growth in chicken [27]. Three significant SNPs were located specifically within the *BMP7* gene. Notably, the significant signals dropped below a significant threshold when the statistical model adjusting for body weight was applied (Fig. 4b), while the strength of the associations increased significantly (*p* < 2.84 × 10^−6^) when the model was not corrected for body weight (Fig. 4a).

**Fig. 5:**
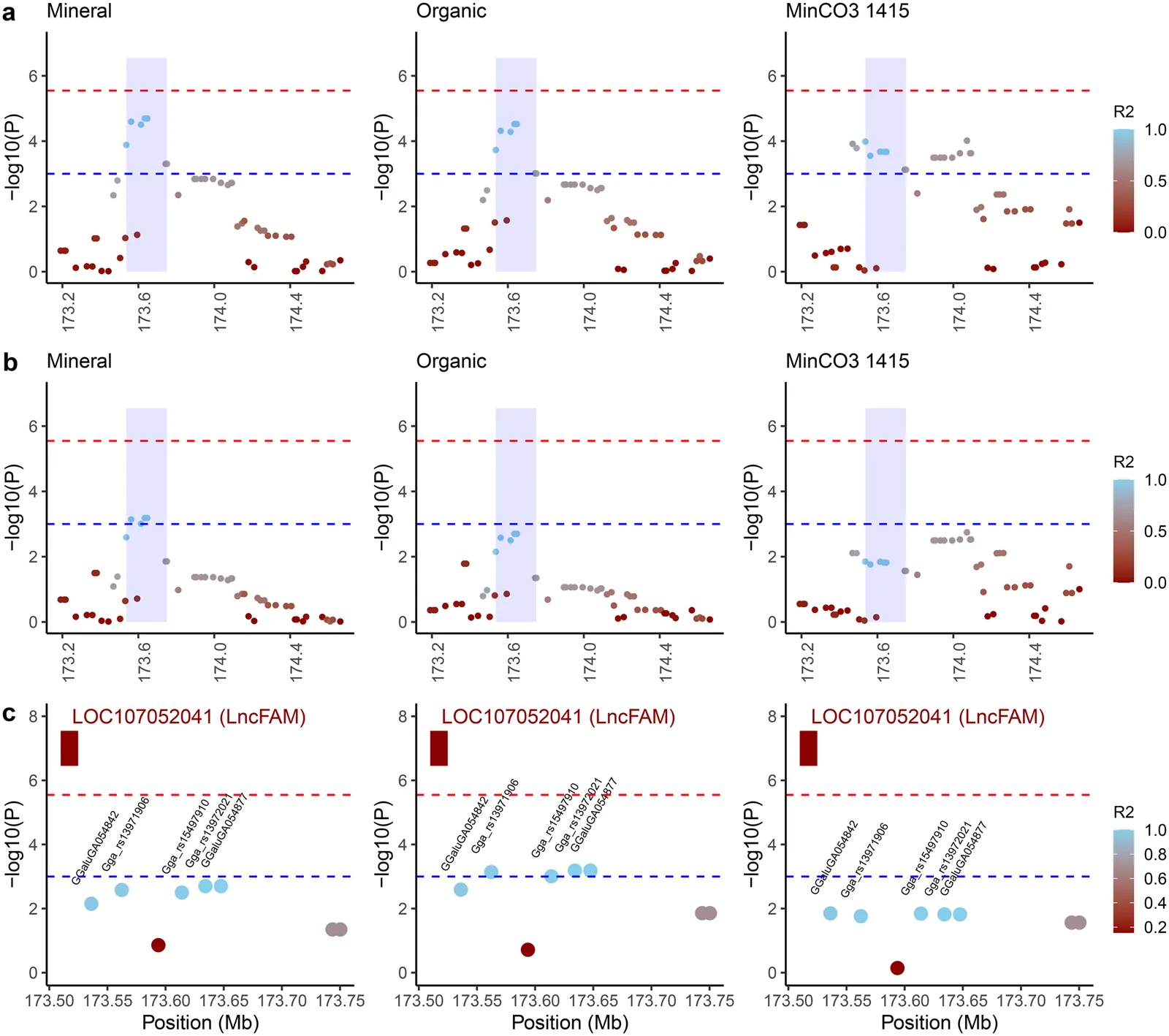
Local linkage disequilibrium (LD) structure around the suggestive SNPs within the chromosome 1 QTL region for medullary organic, mineral, and MinCO3 1415 contents. Plot (**a**) shows results without adjusting for body weight, while plot (**b**) presents the results adjusted for body weight. (**c**) Zoom-in profile of the GWAS results surrounding the LOC107052041 (*LncFAM)* gene position at chromosome 1: 172,01- 172,02 Mb with the vertical rectangles indicating the coding sequence regions of the gene. Each dot represents an SNP marker, colored in accordance with their LD from the top SNP. The shaded rectangular highlights the region of interest at chromosome 1: 173.5–173.6 Mb. The red and blue lines indicate the Bonferroni significance (*p* = 2.84 × 10^−6^) and suggestive association (*p* = 1 × 10^−3^) thresholds

A similar pattern was observed at the suggestive QTL on Chr1: 173.5–173.6 Mb that contains the long non-coding RNA *LOC107052041* (*LncFAM*; Additional file 2, Table S1). This gene has previously been located within QTLs for growth-related traits, and polymorphisms in *LncFAM* have been associated with body weight and skeletal dimensions in chickens [28, 29]. We therefore examined this region using models with and without body weight as a covariate. As in the case of the *BMP7* region, adjustment for body weight decreased the strength of the association signal with medullary mineral and organic contents from *p* < 1 × 10^−5^ to *p* < 1 × 10^−3^ (Fig. 5a,b).

### Phenotypic correlations

The phenotypic correlations for bone mechanical and density parameters and tibial morphometric traits are given in Additional file 1, Figure S7a, and they were consistent with the previously published data [11]. The phenotypic correlations for the studied compositional traits are given in Additional file 1, Figure S9 and Tables S5. Generally, high coefficients amongst either TGA or FTIR traits were consistent with genetic correlations in cortex and medulla. Phenotypic correlations for the most heritable traits, including mineral and organic contents, like the estimates of genetic correlations, were high and significant. In medulla, the correlation between mineral and organic contents was −0.97 (*p* < 0.05), and in cortex it was −0.71 (*p* < 0.05).

### Genomic heritabilities and correlations

#### Bone mechanical and density traits

Consistent with previous pedigree-based estimates, the heritability estimates for bone mechanical and density parameters in tibia bone varied from 0.10 ± 0.04 to 0.33 ± 0.05, with the highest estimates for tibia density (Table 1). All these traits were highly genetically correlated with coefficients ranging from 0.72 ± 0.01 to 0.99 ± 0.04 (Additional file 1, Figure S7b; Additional file 2, Table S2). Heritabilities for humeri bone mechanical and density records were lower and ranged between 0.10 ± 0.04 and 0.29 ± 0.05, with the most heritable being humeri stiffness (Table 1). The genetic correlation between humeri traits varied widely (0.01±0.2 -0.90±0.06), with the strongest correlation observed between humeri breaking strength and stiffness (Additional file 1, Figure S7b; Additional file 2, Table S2). Amongst the tibia and humerus traits, the strongest genetic correlations were observed between humerus breaking strength and stiffness with tibia breaking strength, stiffness, and density (0.44±0.12 - 0.69 ± 0.11; Additional file 1, Figure S7b). Finally, the genomic heritability for the keel bone radiographic density, as in the case with previously reported pedigree estimates [11], was low (0.08±0.04; Table 1).

#### Bone material chemical properties

Consistent with the previously published pedigree-based estimates [11], heritabilities for TGA traits (0.01±0.01 to 0.19 ± 0.07) were higher compared to FTIR traits (0.001±0.0 to 0.12 ± 0.07; Table 2). TGA mineral and organic contents were found to be the most heritable traits (~0.19±0.07) in medulla, and the estimates were higher than in cortex (0.09±0.04). Similarly, the highest heritability among FTIR traits was observed for sulfate content in medullary bone (0.12±0.07). The remaining traits had heritability estimates below 0.07. In addition, the strongest genetic correlations (−0.9) were observed between TGA mineral and organic contents in cortical and medullary bones, with standard errors being lower for these traits in the medulla (SE: 0.18) compared to the cortex (SE: 0.33; Additional file 1, Figure S10; Additional file 2, Table S5). The genetic correlations among FTIR traits in both cortical and medullary bones for most of the parameters were high; however, those had large standard errors (Additional file 1, Figure S10; Additional file 2, Table S5).

Estimated genetic correlations between bone mechanical and density traits and tibial morphometric traits, and cortical and medullary bone composition traits, are shown in Additional file 1, Figure S11. A particular attention was given to the genetic correlations between bone compositional traits and tibia density (Table 2), which emerged as the most heritable trait (h^2^ = 0.33 ± 0.05). As with the pedigree-based estimates [11], the highest and significant correlations of tibia density were observed with TGA traits mineral (0.72 ±0.08) and organic (−0.7 ±0.08) contents in medulla as compared to these records in cortex that were lower and with higher standard errors (organic: −0.48±0.26; mineral: 0.37 ± 0.2; Table 2). Genetic correlations of the tibia density with FTIR traits in both cortex and medulla varied highly and were associated with high standard errors (Table 2).

## Discussion

In this study, we combined bone mechanical, density, morphometric, and composition traits with genotype data to explore the genetics underlying bone characteristics in the commercial line of White Leghorn laying hens. We focused particularly on the significant and suggestive genetic associations with tibial cortical and medullary bone composition traits, followed by the analysis of their heritability estimates and genetic correlations with bone mechanical and density traits of the tibia and humerus.

### Genetic associations with compositional traits in medulla

Seven SNPs within the QTL at Chr1: 173.5–173.6 Mb showed overlapping suggestive associations with two TGA (organic and mineral contents) and with one FTIR (total amount of carbonate in bone mineral MinCO3 1415) traits in tibia medullary bone (Additional file 2, Table S1). The estimated SNP effects for organic and mineral content were in opposite directions, suggesting that this QTL region may influence the relative balance between the mineral and organic components of medullary bone. This QTL region on chromosome 1 harbored seven candidate genes, two out of which (*ALG5* and *SMAD9)* have functions related to bone traits. Because direct functional evidence in chickens is limited for some of the identified positional candidate genes, evidence from their human and other mammalian orthologues was used to assess their biological plausibility. *ALG5*, for example, encodes an enzyme which is involved in the glycosylation process [30] and is known to be involved in regulation of cell functions in the bone marrow micro-environment [31]. *SMAD9* gene plays a role in the bone morphogenetic protein signaling pathway [32] and is crucial for bone development and maintenance [33]. Mutation in *SMAD9* gene leads to increased activity of osteoblast and high bone mass formation [33]. Because of linkage disequilibrium, these associations represent a large region (~144 kb) of highly correlated markers (*r*^*2*^ > 0.8 from a lead marker at 173,647,596 bp) that can span multiple genes. It overlaps with *ALG5* and *SMAD9* genes, as well as other coding and non-coding elements that require further investigation. Notably, one gene within this region, LOC107052041 (*LncFAM,* 172,014,765 - 172,025,976 bp, GenBank assembly GCF 016699485.2, annotation release 106), was previously located within QTLs for growth, feed intake, and feed efficiency in a White Leghorn and Dongxiang reciprocal cross population [28, 29]. In addition, polymorphisms in *LncFAM* have been associated with body weight and skeletal dimensions in chickens, and the gene has been proposed to regulate its target gene, *SUPT20H*. Interestingly, *SUPT20H* was also detected in the current study within the QTL at Chr1: 173.5–173.6 Mb, and it is required for normal axial skeletal development in mice [34], providing an additional biological support for a possible role of this regulatory pathway in skeletal traits. Moreover, the association signals for medullary mineral and organic contents within this region dropped as compared to the unadjusted model (Fig. 5). Together with the previously reported associations of *LncFAM* with growth-related traits, this suggests that this associations may partly reflect shared genetic effects on body weight and medullary bone composition.

Among the other positional candidate genes, *EXOSC8* is a core component of the RNA exosome involved in RNA processing and degradation [35], and *RFXAP* regulates MHC class II gene transcription [36], whereas *SERTM1* remains poorly characterized. Their potential roles in bone composition remain unclear. Given the current genomic resolution, the causal genes cannot be pinpointed; therefore, replication, fine-mapping, and bone-specific expression analyses are required to resolve this locus and establish the underlying mechanism.

### Genetic associations with compositional traits in the cortex

The genetic analysis of cortical compositional traits did not result in significant genome-wide associations. Their low genomic heritability in this population (*h*^*2*^ < 0.09) indicates limited additive genetic variance, which, together with the sample size (*n* ≈ 900), reduced the power to detect variants with small effects. Instead, we highlighted several regions on Chr 3, Chr 19, and Chr 28 with suggestive associations for the relative amount of mineral to organic matrix (CO3 1415/Amide I and PO4/Amide I), with the highest number of overlapped SNPs on Chr 19 (0.13–0.51 Mb). This region harbored 48 genes, including FZD9 (involved in osteoblast formation and matrix mineralization in mice [37]), TNXB (its deficiency is involved in bone loss in mice [38]), and ANGPTL2/ANGPTL1 (involved in osteoclast formation and bone remodeling in mouse cells [39]). We also detected a region on Chr 10: 18.53–18.68 Mb (Additional file 2, Table S1, Additional file 1, Figure S5) displaying suggestive association for the FTIR relative amount of secondary carbonate to the primary carbonate content (CO3 870/CO3 1415) and amount of secondary carbonate substitution in the cortex mineral (MinCO3 870). These components could be related to the processes of bone remodeling and mineralization, where the substitution of carbonate in the bone cortex might influence the overall stability and mechanical properties of the bone tissue [15, 40]. This region contains 11 genes, including *RAB11A*, which is involved in bone biology due to its role in promoting osteoblastic differentiation [41]. However, these FTIR traits showed low genetic variation (*h*^*2*^ < 0.01), likely due to greater analytical variability of the FTIR method compared with TGA. Consequently, the high genetic correlations estimated between FTIR-derived traits and tibial mechanical and density parameters (0.99; Table 2) should be questionable, as genetic correlations involving traits with near-zero heritability may be estimated incorrectly.

### Genetic associations with bone mechanical, density and morphometric traits

We identified genomic regions strongly associated humeri bone stiffness (chromosome 20). The same genetic locus associated with humeral stiffness (Chr 20: 11.55–12.14 Mb) also shows a suggestive association with humeral breaking strength and tibial cortical thickness. Given the overlapped associations and weak to moderate positive phenotypic correlations (~0.15, *p* < 0.05) as well as genetic correlations (0.29±0.28 - 0.64 ± 0.06), we hypothesize that humeri breaking strength is related to the thickness of the cortical bone. Furthermore, we observed that individuals with the G/G genotype for the top significant SNP at 11,715,985 bp exhibited a significantly higher phenotype. The top SNP is highly correlated with 13 surrounding SNPs, suggesting that our study has identified a set of variants that significantly influence humeral breaking strength and cortical thickness. As in the case with to the signals for the medullary mineral and organic contents at QTL on Chr1: 173.5–173.6 Mb, when the GWAS analysis was adjusted for body weight as a covariate, the association signal dropped below the significance threshold (*p* > 2.84 × 10^−6^, Fig. 4a). These two models represent different biological questions: the unadjusted analysis captures the total association with breaking strength, including effects shared with or mediated through body weight, whereas the adjusted analysis evaluates associations with breaking strength independently of body weight. At the same time, this region with the correlated SNPs harbors the *bone morphogenetic protein 7* (*BMP7*) gene, which is crucial for skeletal development [25–27] but also associated with body weight in chicken [42]. The highlighted region overlaps with the previously identified QTL on chromosome 20 for bone biomechanical strength in White Leghorn and wild-type red jungle fowl chickens [43]. Additionally, a QTL in a similar region on chromosome 20 has been linked to body weight in Virginia chicken lines [44]. This finding suggests two possible explanations: the observed pattern results from a causal relationship between body weight and bone strength, or the locus may independently influence both traits. However, the present analyses cannot distinguish between these mechanisms. The moderate genetic correlation between body weight and humeral breaking strength (0.39 ±0.14) indicates that part of their association arises from shared genetic background affecting overall growth and skeletal robustness. Genes promoting larger body size are likely to also enhance bone strength due to mechanical-loading adaptation, where heavier birds require and develop stronger humeri to support increased mass [45, 46]. Similar patterns have been reported in Lohman Brown laying hens (74 weeks of age), where heavier birds exhibit greater tibial breaking strength [47], reflecting coordinated genetic control of body size and bone mechanical properties [45, 48].

Interestingly, the association on chromosome 20 observed in our study was not detected in GWAS for humeral breaking strength conducted in Rhode Island Red laying hens [10]. This discrepancy may be attributed to differences in genetic architecture between the populations, such as variations in allele frequencies or line-specific selection pressures influencing bone strength and body weight traits. Similarly, while our analysis did not identify significant associations for cortical lipids, such associations were reported in Rhode Island Red chickens [10]. This also highlights the importance of studying diverse genetic lines to uncover the genetics of complex bone traits.

### Bone composition heritabilities and genetic correlations

Among the compositional traits, medullary TGA organic and mineral content had the highest genomic heritabilities (0.19 ± 0.05), which aligns with the previously estimated pedigree-based heritabilities (0.14–0.18 [49]). These traits have inverse relationship which is captured by the negative genetic correlation (−0.99, *p* < 0.01), highlighting the inherent balance between the proportion of organic and mineral matrices in bone composition. Furthermore, they are strongly genetically correlated with tibia density (0.7 ± 0.08), showing a positive sign with mineral and negative sign with organic content. These genetic correlations align with phenotypic correlations (Additional file 1, Figure S11), suggesting that higher mineral content in the medullary bone contributes to improved bone density properties. Our data is consistent with the previously published results in Rhode Island Red laying hens [10, 11] and the pedigree-based estimates in White Leghorn laying hens [11].

One of the key traits of interest for improving in the commercial laying hens using selective breeding is keel bone [6]. The present analysis was based on the available X-ray density phenotype and found low heritability as well as no significant markers were detected through GWAS. These results align with the pedigree-based data [11], suggesting that alternative approaches are required to phenotype this trait. One of the latest studies reported several new characteristics like keel bone depth and area of the lateral surface with moderate heritabilities (0.40) [50]. In another study [51], it was shown that the analysis of x-ray images of keel in hens can be improved using a deep learning model with a prospective result for genetic improvements. These approaches provide valuable directions for future genetic studies of keel bone characteristics.

Most bone material traits measured with the TGA method that had high genetic correlations with tibia density were associated with the medullary bone rather than the cortical bone (Table 2). Although these estimates had high standard errors, they aligned with pedigree-based findings [11], showing the contribution of the tibia medullary bone composition to the density of the bone. Several FTIR compositional traits related to cortical (PO4/Amide I, MinCO3 870, CO3/Amide I, CO3 870/CO3 1415) and medullary mineralization (CO3/Amide I, CO3 870/1415 and crystallinity index) traits exhibited exceptionally high correlations (0.87±1.44 - 0.99 ± 0.0) with tibia density. Dunn et al. [11] also revealed high genetic correlations (0.69 ± 0.18 to 0.90 ± 0.06) with tibia density for the same low heritably traits in the medulla (e.g., PO4/Amide I and CO3/Amide I). In the line with previous studies, the medullary bone mineral component has a crucial contribution to bone properties, including fracture resistance [52] or breaking strength [3, 53]. However, the low heritability of the FTIR traits (genomic *h*^*2*^ < 0.07) indicates limited additive genetic variance, which can result in unstable genetic covariance and correlation estimates. Consequently, these estimates should not be interpreted as evidence of a nearly perfect genetic relationship with tibia density. Because FTIR measurements are more variable than TGA measurements [10], technical replication, standardized sample preparation, and data processing could improve measurement precision of the FTIR traits.

Finally, two new phenotypes were analyzed in this work, including cortical diameter and thickness of the tibia bone. Tibia cortical thickness has been previously reported to differ in hens housed in different systems (e.g., in floor pens and in furnished cages [54]). The heritability estimates for these parameters were low (diameter: 0.16 ± 0.05; thickness: 0.10 ± 0.04). However, the genetic correlation between cortical diameter and tibia density was moderate and significant (0.29±18 - 0.45 ± 0.27), suggesting a shared genetic basis between these traits.

## Conclusions

This study investigated the genetic architecture of tibial cortical and medullary bone composition traits and estimated their genetic relationship with bone mechanical and density traits, and tibial morphometric characteristics. A set of markers that significantly influence humeral bone stiffness, strength and cortical thickness was highlighted, located within the previously identified QTL on chromosome 20 for bone biomechanical strength and body weight. This region contains *BMP7*, a strong positional candidate because of its established role in skeletal development and its reported association with body weight in chickens. We also identified a suggestive QTL on chromosome 1 (173.5–173.6 Mb), with the strongest associations observed for medullary mineral and organic contents. This region contains the long non-coding RNA *LOC107052041 (LncFAM)*, polymorphisms have been previously observed to be associated with body weight and skeletal dimensions in chickens. Together, these findings highlight two candidate genomic regions for bone strength (significant effect) and composition (suggestive effect) and suggest a possible shared genetic basis between skeletal and growth-related traits. Finally, more accurate heritabilities were estimated for TGA traits as compared to FTIR traits, with medullary organic and mineral contents being the most heritable and highly correlated with tibia density traits. Overall, these findings identified genomic loci associated with variation in bone quality traits, providing candidates for functional validation and for future evaluation of their potential to improve prediction of bone strength in laying hens.

## Electronic supplementary material

Below is the link to the electronic supplementary material.

Supplementary Material 1

Supplementary Material 2

Supplementary Material 3

## Data Availability

The datasets analysed during the current study containing genotypes information are not publicly available due to commercial sensitivity.
