# Supplementary material for: Genetic architecture of bone strength and composition in White Leghorn laying hens
Source: Genet Sel Evol. 2026 Nov 3;58:80. doi: 10.1186/s12711-026-01103-3 (PMC13733021; doi:10.1186/s12711-026-01103-3)
Supplement: Supplementary file 1 [file 12711_2026_1103_MOESM1_ESM.docx]

**Additional file 1**

**Genetic architecture of bone strength and composition in White Leghorn laying hens**

Khrystyna Kurta^1^, Peter W. Wilson^2^, Björn Andersson^3^, Matthias Schmutz^3^, David Cavero^4^, Tattiana Enrich-Essvein^5^, Laura Crespo-Lopez^5^, Alejandro B. Rodriguez‑Navarro^5^, Ian C. Dunn^2^, Dirk‑Jan de Koning^1^ & Martin Johnsson^1^

^1^Swedish University of Agricultural Sciences, Uppsala, Sweden;

*^2^Roslin Institute, University of Edinburgh, Scotland, UK;*

*^3^Lohmann Breeders, Cuxhaven, Germany;*

*^4^H&N International, Cuxhaven, Germany*

*^5^Departamento de Mineralogia y Petrologia, Universidad de Granada, Granada, Spain;*

Table of Contents

[Figure S1 Phenotypic distributions of mechanical and density traits of the tibia and humerus, morphometric traits of the tibia, and keel radiographic density traits in White Leghorn laying hens.	3](#_Toc243906233)

[Figure S2 Phenotypic distributions of tibial cortical bone composition traits measured by thermogravimetry and infrared spectroscopy in White Leghorn laying hens.	4](#_Toc243906234)

[Figure S3 Phenotypic distributions of tibial medullary bone composition traits measured by thermogravimetry and infrared spectroscopy in White Leghorn laying hens.	5](#_Toc243906235)

[Figure S4 Manhattan plot (left), showing the −log10(P) for each SNP for bone mechanical traits. The red line indicates the Bonferroni significance threshold of P=2.84 ×10^−6^, and the blue line represents the suggestive significance threshold of P =1 ×10^-3^.	6](#_Toc243906236)

[Figure S5 Manhattan plots, showing the −log10(P) for each SNP for the tibia cortical bone composition parameters measured by thermogravimetry and infrared spectroscopy. The red line indicates the Bonferroni significance threshold of P =2.84 ×10^−6^, and the blue line represents the suggestive significance threshold of P =1 ×10^-3^.	7](#_Toc243906237)

[Figure S6 Manhattan plots, showing the −log10(P) for each SNP for the tibia medullary bone composition parameters measured by thermogravimetry and infrared spectroscopy. The red line indicates the Bonferroni significance threshold of 2.84 ×10^−6^, and the blue line represents the suggestive significance threshold of P =1 ×10^-3^.	8](#_Toc243906238)

[Figure S7 (a) Phenotypic and (b) genetic correlations among bone mechanical and density traits of the tibia, humerus, and keel and tibial morphometric traits. For phenotypic correlations, asterisks indicate P < 0.05*, P < 0.01**, P < 0.001***. For genetic correlations, asterisks indicate P < 0.1*, P < 0.05**, P < 0.01***.	9](#_Toc243906239)

[Figure S8 (a) LD plots showing the local LD structure around the most significant SNPs (55,553,280; 55,598,304; and 55,662,347 bp) in the QTL regions on chromosome 1, 1Mb upstream and downstream.	10](#_Toc243906240)

[Figure S9 Phenotypic correlations among tibial cortical and medullary bone composition traits measured by thermogravimetry and infrared spectroscopy.	11](#_Toc243906241)

[Figure S10 Estimated genetic correlations among the tibia cortical and medullary bone composition traits measured by thermogravimetry and infrared spectroscopy.	12](#_Toc243906242)

[Figure S11 Phenotypic (top) and estimated genetic (bottom) correlations between whole-bone mechanical and density traits and tibial morphometric traits, and tibial cortical and medullary bone composition traits measured by thermogravimetry and infrared spectroscopy.	13](#_Toc243906243)

# Figure S1 Phenotypic distributions of mechanical and density traits of the tibia and humerus, morphometric traits of the tibia, and keel radiographic density traits in White Leghorn laying hens.

# Figure S2 Phenotypic distributions of tibial cortical bone composition traits measured by thermogravimetry and infrared spectroscopy in White Leghorn laying hens.

# **Figure S3** Phenotypic distributions of tibial medullary bone composition traits measured by thermogravimetry and infrared spectroscopy in White Leghorn laying hens.

# Figure S4 Manhattan plot (left), showing the −log10(P) for each SNP for bone mechanical traits. The red line indicates the Bonferroni significance threshold of *P*=2.84 ×10^−6^, and the blue line represents the suggestive significance threshold of *P* =1 ×10^-3^.

# Figure S5 Manhattan plots, showing the −log10(P) for each SNP for the tibia cortical bone composition parameters measured by thermogravimetry and infrared spectroscopy. The red line indicates the Bonferroni significance threshold of *P* =2.84 ×10^−6^, and the blue line represents the suggestive significance threshold of *P* =1 ×10^-3^.

# Figure S6 Manhattan plots, showing the −log10(P) for each SNP for the tibia medullary bone composition parameters measured by thermogravimetry and infrared spectroscopy. The red line indicates the Bonferroni significance threshold of 2.84 ×10^−6^, and the blue line represents the suggestive significance threshold of *P* =1 ×10^-3^.

# **Figure S7** (a) Phenotypic and (b) genetic correlations among bone mechanical and density traits of the tibia, humerus, and keel and tibial morphometric traits. For phenotypic correlations, asterisks indicate *P* < 0.05*, *P* < 0.01**, *P* < 0.001***. For genetic correlations, asterisks indicate *P* < 0.1*, *P* < 0.05**, *P* < 0.01***.

Figure S8 (a) LD plots showing the local LD structure around the most significant SNPs (55,553,280; 55,598,304; and 55,662,347 bp) in the QTL regions on chromosome 1, 1Mb upstream and downstream. Each dot represents an SNP marker. The y-axis indicates the significance of the SNP (-log10(*P*)-value), and the color indicates the level of LD with the top SNP. The shaded rectangular highlights the region of interest at Chr 1: 55.55-79.83 Mb. **(b)** The minor allele frequencies in the chromosome region are depicted under the x-axis. **(c)** Boxplot showing the effect of one of the most significant SNPs (Chr 1: 55,598,304 bp) on tibia bone breaking strength, stiffness and density. Wilcoxon rank test *P*-values: * *P*<0.05, **p<0.01, ***p<0.001.

Figure S9 Phenotypic correlations among tibial cortical and medullary bone composition traits measured by thermogravimetry and infrared spectroscopy. Asterisks indicate *P*<0.05*, *P*<0.01**, *P*<0.001***. Measurement methods and trait definitions are provided in Table 2 of the main text.

Figure S10 Estimated genetic correlations among the tibia cortical and medullary bone composition traits measured by thermogravimetry and infrared spectroscopy. For methods and the respective trait description, see Table 2 in the main text.

Figure S11 Phenotypic (top) and estimated genetic (bottom) correlations between whole-bone mechanical and density traits and tibial morphometric traits, and tibial cortical and medullary bone composition traits measured by thermogravimetry and infrared spectroscopy. For phenotypic correlations, asterisks indicate *P*<0.05*, *P*<0.01**, *P*<0.001***. Measurement methods and trait definitions are provided in Table 2 of the main text.
