# Supplementary material for: Genetic architecture of bone strength and composition in White Leghorn laying hens
Source: Genet Sel Evol. 2026 Nov 3;58:80. doi: 10.1186/s12711-026-01103-3 (PMC13733021; doi:10.1186/s12711-026-01103-3)
Supplement: Supplementary file 2 [file 12711_2026_1103_MOESM2_ESM.docx]

**Additional file 2**

**Genetic architecture of bone strength and composition in White Leghorn laying hens**

Khrystyna Kurta^1^, Peter W. Wilson^2^, Björn Andersson^3^, Matthias Schmutz^3^, David Cavero^4^, Tattiana Enrich-Essvein^5^, Laura Crespo-Lopez^5^, Alejandro B. Rodriguez‑Navarro^5^, Ian C. Dunn^2^, Dirk‑Jan de Koning^1^ & Martin Johnsson^1^

^1^Swedish University of Agricultural Sciences, Uppsala, Sweden;

*^2^Roslin Institute, University of Edinburgh, Scotland, UK;*

*^3^Lohmann Breeders, Cuxhaven, Germany;*

*^4^H&N International, Cuxhaven, Germany*

*^5^Departamento de Mineralogia y Petrologia, Universidad de Granada, Granada, Spain;*

Table of Contents

[Table S1 Genetic markers with significant and suggestive associations overlapped across the bone strength and compositional traits in White Leghorn laying hens.	2](#_Toc243906508)

[Table S2 Estimates of genetic correlations (below the diagonal), and phenotypic correlations (above the diagonal) with standard errors (in brackets) for the bone mechanical, density traits, and morphometric traits.	6](#_Toc243906509)

[Table S3 Genes (n = 255) within genetic markers with overlapped significant and suggestive associations (Table S1) with bone strength and compositional traits in White Leghorn laying hens.	7](#_Toc243906510)

[Table S4 Known functions of genes located within 100 kb upstream or downstream of significant SNPs (P < 1 ×10^-5^) identified in the GWAS.	9](#_Toc243906511)

# **Table S1** Genetic markers with significant and suggestive associations overlapped across the bone strength and compositional traits in White Leghorn laying hens.

| Traits | Chr | SNP | Position | A1 | SNP effect | -log10(P)-value |
| --- | --- | --- | --- | --- | --- | --- |
| Tibia stiffness, Tibia density | 1 | GGaluGA018750 | 55553280 | G | 1.38, 0.96 | 5.6, 3 |
| Tibia stiffness, Tibia density | 1 | GGaluGA018772 | 55598304 | A | 1.38, 0.96 | 5.6, 3 |
| Tibia stiffness, Tibia density | 1 | Gga_rs13867977 | 55662347 | G | 1.38, 0.96 | 5.6, 3 |
| Tibia stiffness, Tibia density | 10 | GGaluGA072797 | 21042272 | A | -0.22, -0.21 | 3.3, 3.1 |
| Tibia stiffness, Tibia density | 10 | GGaluGA072861 | 21205889 | A | -0.24, -0.23 | 3.7, 3.4 |
| Tibia stiffness, Tibia density | 10 | Gga_rs14954427 | 21182061 | A | -0.23, -0.23 | 3.6, 3.5 |
| Tibia density, Hum breaking strength | 3 | Gga_rs15395200 | 78898678 | C | 0.2, 0.2 | 3.6, 3.6 |
| Tibia breaking strength, Tibia stiffness | 5 | GGaluGA291009 | 58459769 | G | 0.27, 0.31 | 3.9, 5.1 |
| Tibia breaking strength, Tibia density | 3 | GGaluGA229689 | 79438685 | G | 0.2, 0.21 | 3.4, 3.8 |
| Tibia breaking strength, Tibia density | 3 | Gga_rs14380197 | 79410376 | A | 0.2, 0.21 | 3.4, 3.8 |
| Tibia breaking strength, Tibia density | 3 | Gga_rs14380390 | 79546262 | A | 0.2, 0.22 | 3.4, 3.9 |
| Tibia breaking strength, Tibia density | 3 | Gga_rs15396978 | 79452214 | G | 0.2, 0.21 | 3.4, 3.8 |
| Tibia breaking strength, Tibia density | 3 | Gga_rs15397501 | 79578304 | G | 0.2, 0.22 | 3.3, 4 |
| Tibia breaking strength, Tibia density | 22 | Gga_rs10722068 | 817162 | A | -0.19, -0.19 | 3.2, 3.3 |
| Tibia breaking strength, Tibia density | 22 | Gga_rs13725325 | 811524 | A | -0.19, -0.19 | 3.2, 3.3 |
| Tibia breaking strength, Tibia density | 22 | Gga_rs15187476 | 746031 | G | -0.18, -0.19 | 3.2, 3.3 |
| Tibia breaking strength, Tibia density | 22 | Gga_rs15187583 | 932152 | G | -0.18, -0.19 | 3.1, 3.3 |
| Hum stiffness, Tibia stiffness | 1 | GGaluGA042705 | 128422755 | A | 0.21, 0.22 | 3.2, 3.8 |
| Hum stiffness, Tibia cortical thickness, Hum breaking strength | 20 | GGaluGA181109 | 11849437 | A | -0.3, -0.21, -0.26 | 5.3, 3.7, 4 |
| Hum stiffness, Tibia cortical thickness, Hum breaking strength | 20 | GGaluGA181122 | 11874967 | A | -0.3, -0.21, -0.26 | 5.3, 3.7, 4 |
| Hum stiffness, Tibia cortical thickness, Hum breaking strength | 20 | GGaluGA181132 | 11894308 | A | -0.3, -0.21, -0.24 | 5.2, 3.5, 3.6 |
| Hum stiffness, Tibia cortical thickness, Hum breaking strength | 20 | GGaluGA181137 | 11896159 | A | -0.3, -0.21, -0.24 | 5.2, 3.5, 3.6 |
| Hum stiffness, Tibia cortical thickness, Hum breaking strength | 20 | GGaluGA181198 | 12001707 | G | -0.3, -0.21, -0.24 | 5.2, 3.5, 3.6 |
| Hum stiffness, Tibia cortical thickness, Hum breaking strength | 20 | GGaluGA181268 | 12140945 | G | -0.28, -0.2, -0.22 | 4.7, 3.4, 3.1 |
| Hum stiffness, Tibia cortical thickness, Hum breaking strength | 20 | Gga_rs10730825 | 11980848 | G | -0.3, -0.2, -0.24 | 5.2, 3.5, 3.6 |
| Hum stiffness, Tibia cortical thickness, Hum breaking strength | 20 | Gga_rs14279984 | 11867050 | G | -0.3, -0.21, -0.26 | 5.3, 3.7, 4 |
| Hum stiffness, Tibia cortical thickness, Hum breaking strength | 20 | Gga_rs14280180 | 12016410 | A | -0.3, -0.21, -0.24 | 5.2, 3.5, 3.6 |
| Hum stiffness, Tibia cortical thickness, Hum breaking strength | 20 | Gga_rs15179074 | 11828660 | G | -0.31, -0.21, -0.26 | 5.5, 3.7, 4.1 |
| Hum stiffness, Tibia cortical thickness, Hum breaking strength | 20 | Gga_rs15179147 | 11957957 | G | -0.3, -0.21, -0.24 | 5.2, 3.5, 3.6 |
| Hum stiffness, Tibia cortical thickness, Hum breaking strength | 20 | Gga_rs15179488 | 12290992 | G | -0.26, -0.2, -0.22 | 4.1, 3.3, 3 |
| Hum stiffness, Tibia cortical thickness, Hum breaking strength | 20 | Gga_rs16174634 | 11947699 | A | -0.3, -0.21, -0.24 | 5.2, 3.5, 3.6 |
| Hum stiffness, Tibia cortical thickness, Hum breaking strength | 20 | Gga_rs16174733 | 11999300 | G | -0.3, -0.2, -0.24 | 5.3, 3.5, 3.6 |
| Hum stiffness, Tibia cortical thickness, Hum breaking strength | 20 | Gga_rs16174986 | 12114882 | A | -0.29, -0.2, -0.24 | 5, 3.3, 3.5 |
| Hum stiffness, Tibia cortical thickness | 20 | Gga_rs14280350 | 12151445 | A | -0.27, -0.2 | 4.4, 3.3 |
| Hum stiffness, Medullary_MinCO3_1415 | 20 | Gga_rs16174090 | 11551097 | A | 0.2, 0.18 | 3, 3.2 |
| Hum stiffness, Hum breaking strength | 1 | Gga_rs15415601 | 129311997 | A | -0.23, -0.24 | 3.2, 3.4 |
| Hum stiffness, Hum breaking strength | 5 | Gga_rs16458857 | 475797 | A | 0.24, 0.28 | 3, 4.1 |
| Hum stiffness, Hum breaking strength | 20 | GGaluGA180980 | 11591655 | A | -0.33, -0.27 | 6.6, 4.6 |
| Hum stiffness, Hum breaking strength | 20 | GGaluGA181090 | 11781599 | A | -0.34, -0.27 | 6.9, 4.7 |
| Hum stiffness, Hum breaking strength | 20 | Gga_rs13635060 | 11613151 | G | -0.32, -0.27 | 6.5, 4.7 |
| Hum stiffness, Hum breaking strength | 20 | Gga_rs13635072 | 11659437 | A | -0.33, -0.25 | 6.7, 4.2 |
| Hum stiffness, Hum breaking strength | 20 | Gga_rs13635266 | 12090265 | A | 0.3, 0.26 | 5.1, 3.9 |
| Hum stiffness, Hum breaking strength | 20 | Gga_rs14279707 | 11644500 | G | -0.33, -0.27 | 6.6, 4.6 |
| Hum stiffness, Hum breaking strength | 20 | Gga_rs14280220 | 12061503 | G | 0.3, 0.26 | 5.2, 3.9 |
| Hum stiffness, Hum breaking strength | 20 | Gga_rs15178814 | 11614990 | G | -0.33, -0.27 | 6.5, 4.6 |
| Hum stiffness, Hum breaking strength | 20 | Gga_rs15178951 | 11715985 | A | -0.34, -0.26 | 7, 4.5 |
| Hum stiffness, Hum breaking strength | 20 | Gga_rs15178994 | 11772647 | G | 0.32, 0.26 | 6.5, 4.5 |
| Hum stiffness, Hum breaking strength | 20 | Gga_rs15179186 | 12013159 | G | 0.31, 0.26 | 5.4, 3.9 |
| Hum stiffness, Hum breaking strength | 20 | Gga_rs16174156 | 11608099 | A | -0.33, -0.27 | 6.5, 4.6 |
| Hum stiffness, Hum breaking strength | 20 | Gga_rs16174195 | 11627078 | A | -0.33, -0.27 | 6.5, 4.6 |
| Hum stiffness, Hum breaking strength | 20 | Gga_rs16174283 | 11692291 | A | -0.33, -0.26 | 7, 4.5 |
| Hum stiffness, Hum breaking strength | 20 | Gga_rs16174305 | 11707312 | G | -0.33, -0.26 | 6.9, 4.5 |
| Hum stiffness, Hum breaking strength | 20 | Gga_rs16174461 | 11803465 | A | -0.32, -0.27 | 6.6, 4.8 |
| Hum stiffness, Hum breaking strength | 20 | Gga_rs16174933 | 12092729 | G | 0.3, 0.26 | 5.2, 3.9 |
| Hum bone score, Cortical_Water | 17 | GGaluGA112246 | 963988 | G | 0.2, -0.17 | 3.3, 3.1 |
| Hum bone score, Cortical_Water | 17 | GGaluGA112260 | 995536 | A | 0.21, -0.17 | 3.5, 3 |
| Hum bone score, Cortical_Water | 17 | Gga_rs15035176 | 989408 | G | 0.21, -0.17 | 3.5, 3 |
| Cortical_Sulfates, Medullary_CO3_1450.CO3_1415 | 6 | GGaluGA302459 | 25194543 | G | -0.2, 0.21 | 3.2, 3.5 |
| Cortical_Sulfates, Medullary_CO3_1450.CO3_1415 | 6 | Gga_rs13579089 | 25191737 | G | -0.2, 0.21 | 3.2, 3.5 |
| Cortical_Sulfates, Medullary_CO3_1450.CO3_1415 | 6 | Gga_rs14584500 | 25178685 | A | -0.2, 0.21 | 3.2, 3.5 |
| Cortical_PO4.Amide1, Cortical_CO3_1415.Amide1 | 3 | GGaluGA231703 | 85467314 | G | -0.21, -0.22 | 3.1, 3.4 |
| Cortical_PO4.Amide1, Cortical_CO3_1415.Amide1 | 3 | Gga_rs13696194 | 85405574 | G | -0.21, -0.22 | 3, 3.4 |
| Cortical_PO4.Amide1, Cortical_CO3_1415.Amide1 | 3 | Gga_rs14387149 | 85347154 | A | -0.21, -0.22 | 3, 3.4 |
| Cortical_PO4.Amide1, Cortical_CO3_1415.Amide1 | 3 | Gga_rs14387346 | 85535433 | A | -0.21, -0.22 | 3.1, 3.4 |
| Cortical_PO4.Amide1, Cortical_CO3_1415.Amide1 | 19 | GGaluGA123606 | 179885 | A | -0.27, -0.31 | 3.2, 4 |
| Cortical_PO4.Amide1, Cortical_CO3_1415.Amide1 | 19 | Gga_rs13573038 | 517857 | A | -0.28, -0.31 | 3.4, 4 |
| Cortical_PO4.Amide1, Cortical_CO3_1415.Amide1 | 19 | Gga_rs14115155 | 303515 | A | -0.28, -0.31 | 3.4, 4.1 |
| Cortical_PO4.Amide1, Cortical_CO3_1415.Amide1 | 19 | Gga_rs14115288 | 517607 | A | -0.28, -0.31 | 3.4, 4 |
| Cortical_PO4.Amide1, Cortical_CO3_1415.Amide1 | 19 | Gga_rs15043464 | 328538 | A | -0.28, -0.31 | 3.4, 4.1 |
| Cortical_PO4.Amide1, Cortical_CO3_1415.Amide1 | 19 | Gga_rs15833593 | 137076 | G | -0.27, -0.31 | 3.3, 4 |
| Cortical_PO4.Amide1, Cortical_CO3_1415.Amide1 | 28 | GGaluGA201880 | 2066107 | A | 0.58, 0.5 | 4.1, 3.2 |
| Cortical_PO4.Amide1, Cortical_CO3_1415.Amide1 | 28 | GGaluGA201905 | 2092897 | G | 0.58, 0.51 | 4.1, 3.2 |
| Cortical_PO4.Amide1, Cortical_CO3_1415.Amide1 | 28 | Gga_rs13546080 | 2099210 | G | 0.58, 0.51 | 4.1, 3.2 |
| Cortical_MinCO3_870, Cortical_CO3_870.CO3_1415 | 3 | Gga_rs16222534 | 9472154 | G | -0.23, -0.25 | 3.1, 3.5 |
| Cortical_MinCO3_870, Cortical_CO3_870.CO3_1415 | 10 | GGaluGA071775 | 18634246 | G | 0.21, 0.23 | 3.5, 3.8 |
| Cortical_MinCO3_870, Cortical_CO3_870.CO3_1415 | 10 | Gga_rs13785570 | 18683065 | G | 0.21, 0.23 | 3.5, 3.8 |
| Cortical_MinCO3_870, Cortical_CO3_870.CO3_1415 | 10 | Gga_rs14011752 | 18674708 | C | 0.21, 0.23 | 3.5, 3.8 |
| Cortical_MinCO3_870, Cortical_CO3_870.CO3_1415 | 10 | Gga_rs14952054 | 18537051 | A | 0.21, 0.23 | 3.5, 3.8 |
| Cortical_MinCO3_870, Cortical_CO3_870.CO3_1415 | 10 | Gga_rs14952061 | 18552543 | G | 0.21, 0.22 | 3.4, 3.7 |
| Cortical_MinCO3_870, Cortical_CO3_870.CO3_1415 | 10 | Gga_rs15588563 | 18560915 | A | 0.21, 0.23 | 3.5, 3.8 |
| Cortical_MinCO3_1415, Cortical_CO3_870.CO3_1415 | 3 | Gga_rs14314540 | 9671538 | G | -0.17, 0.16 | 3.2, 3 |
| Cortical_MinCO3_1415, Cortical_CO3_870.CO3_1415 | 8 | GGaluGA323331 | 4316225 | A | 0.19, -0.19 | 3, 3.2 |
| Cortical_Collagen maturity, Medullary_Lipids | 4 | Gga_rs13521195 | 51669500 | G | -0.27, 0.27 | 3, 3 |
| Medullary_PO4.Amide1, Medullary_MinCO3_870, Medullary_CO3_870.CO3_1415 | 4 | Gga_rs13577145 | 25894088 | G | 0.3, 0.33, 0.31 | 3.2, 3.8, 3.3 |
| Medullary_PO4.Amide1, Medullary_CO3_1415.Amide1 | 7 | Gga_rs15831051 | 3496015 | A | 0.89, 0.84 | 4.2, 4 |
| Medullary_PO4.Amide1, Medullary_CO3_1415.Amide1 | 7 | Gga_rs15831184 | 3528136 | G | 0.89, 0.84 | 4.2, 4 |
| Medullary_Organic, Medullary_Mineral, Medullary_MinCO3_1415 | 1 | GGaluGA054842 | 173536168 | A | 0.24, -0.24, -0.22 | 3.7, 3.9, 4 |
| Medullary_Organic, Medullary_Mineral, Medullary_MinCO3_1415 | 1 | GGaluGA054877 | 173647596 | G | 0.27, -0.27, -0.22 | 4.5, 4.7, 3.7 |
| Medullary_Organic, Medullary_Mineral, Medullary_MinCO3_1415 | 1 | Gga_rs13971906 | 173562306 | A | 0.26, -0.27, -0.21 | 4.3, 4.6, 3.5 |
| Medullary_Organic, Medullary_Mineral, Medullary_MinCO3_1415 | 1 | Gga_rs13972021 | 173634193 | G | 0.27, -0.27, -0.22 | 4.5, 4.7, 3.7 |
| Medullary_Organic, Medullary_Mineral, Medullary_MinCO3_1415 | 1 | Gga_rs13972107 | 173743591 | G | 0.2, -0.21, -0.18 | 3, 3.3, 3.1 |
| Medullary_Organic, Medullary_Mineral, Medullary_MinCO3_1415 | 1 | Gga_rs13972116 | 173750242 | G | 0.2, -0.21, -0.18 | 3, 3.3, 3.1 |
| Medullary_Organic, Medullary_Mineral, Medullary_MinCO3_1415 | 1 | Gga_rs15497910 | 173613981 | A | 0.26, -0.27, -0.21 | 4.3, 4.5, 3.7 |
| Medullary_Organic, Medullary_Mineral, Medullary_CO3_1450.CO3_1415, Tibia breaking strength, Tibia density | 2 | GGaluGA130608 | 3576550 | G | -0.19, 0.21, -0.18, 0.2, 0.18 | 3.6, 4.2, 3.3, 3.9, 3.3 |
| Medullary_Organic, Medullary_Mineral, Medullary_CO3_1450.CO3_1415, Tibia breaking strength, Tibia density | 2 | Gga_rs14129759 | 3515191 | A | -0.19, 0.21, -0.17, 0.2, 0.18 | 3.6, 4.2, 3.3, 3.8, 3.2 |
| Medullary_Organic, Medullary_Mineral | 2 | GGaluGA141800 | 37816681 | G | 0.22, -0.21 | 3.2, 3.1 |
| Medullary_Organic, Medullary_Mineral | 2 | Gga_rs15085520 | 37863392 | A | 0.22, -0.21 | 3.2, 3.2 |
| Medullary_Organic, Medullary_Mineral | 5 | Gga_rs15738056 | 57086955 | A | -0.39, 0.41 | 3.1, 3.3 |
| Medullary_Organic, Medullary_Mineral | 11 | Gga_rs16740226 | 17182240 | G | -0.18, 0.18 | 3.2, 3.1 |
| Medullary_CO3_1415.Amide1, Tibia density | 10 | GGaluGA072925 | 21340578 | A | -0.19, -0.2 | 3.6, 3.3 |
| Medullary_CO3_1415.Amide1, Medullary_Sulfates | 22 | Gga_rs14684621 | 2488867 | A | 0.19, 0.17 | 3.9, 3.1 |
| Medullary_CO3_1415.Amide1, Medullary_CO3_870.CO3_1415, Tibia stiffness, Tibia density | 10 | GGaluGA072953 | 21368926 | A | -0.19, -0.17, -0.19, -0.2 | 3.8, 3, 3.2, 3.5 |

# **Table S2** Estimates of genetic correlations (below the diagonal), and phenotypic correlations (above the diagonal) with standard errors (in brackets) for the bone mechanical, density traits, and morphometric traits.

|  | Tibia breaking strength | Tibia density | Tibia stiffness | Hum breaking strength | Hum density | Hum stiffness | Hum bone score | Keel RD | Tibia cortical thickness | Tibia cortical diameter |
| --- | --- | --- | --- | --- | --- | --- | --- | --- | --- | --- |
| Tibia breaking strength | NA | 0.72 (0.0) | 0.74 (0.0) | 0.38 (0.0) | 0.10 (0.0) | 0.38 (0.0) | -0.03 (0.5) | 0.17 (0.0) | 0.16 (0.0) | 0.07 (0.0) |
| Tibia density | 0.88 (0.04) | NA | 0.65 (0.0) | 0.32 (0.0) | 0.17 (0.0) | 0.34 (0.0) | 0.08 (0.0) | 0.31 (0.0) | 0.10 (0.0) | 0.14 (0.0) |
| Tibia stiffness | 0.96 (0.04) | 0.89 (0.06) | NA | 0.37 (0.0) | 0.11 (0.0) | 0.39 (0.0) | -0.06 (0.1) | 0.16 (0.0) | 0.22 (0.0) | 0.03 (0.4) |
| Hum breaking strength | 0.48 (0.12) | 0.47 (0.12) | 0.48 (0.13) | NA | 0.35 (0.0) | 0.63 (0.0) | 0.26 (0.0) | 0.09 (0.0) | 0.19 (0.0) | 0.04 (0.3) |
| Hum density | -0.1 (0.26) | 0.22 (0.24) | -0.06 (0.28) | 0.35 (0.26) | NA | 0.09 (0.0) | 0.55 (0.0) | 0.04 (0.2) | -0.05 (0.1) | 0.02 (0.5) |
| Hum stiffness | 0.58 (0.11) | 0.44 (0.12) | 0.69 (0.11) | 0.9 (0.06) | 0.05 (0.26) | NA | -0.01 (0.7) | 0.06 (0.1) | 0.15 (0.0) | 0.04 (0.3) |
| Hum bone score | -0.19 (0.2) | 0.06 (0.19) | -0.21 (0.25) | 0.3 (0.23) | 0.86 (0.29) | 0.01 (0.2) | NA | 0.04 (0.2) | 0.03 (0.4) | 0.02 (0.6) |
| Keel RD | 0.45 (0.3) | 0.46 (0.3) | 0.19 (0.34) | 0.06 (0.34) | 0.31 (0.55) | 0.12 (0.49) | 0.3 (0.44) | NA | 0.00 (0.9) | 0.09 (0.0) |
| Tibia cortical thickness | 0.49 (0.28) | 0.45 (0.27) | 0.64 (0.28) | 0.29 (0.28) | -0.15 (0.45) | 0.64 (0.54) | -0.58 (0.65) | 0.52 (0.72) | NA | -0.07 (0.0) |
| Tibia cortical diameter | 0.24 (0.19) | 0.29 (0.18) | 0.08 (0.22) | 0.15 (0.2) | 0.32 (0.33) | -0.02 (0.2) | 0.24 (0.26) | 0.39 (0.45) | -0.35 (0.39) | NA |

# **Table S3** Genes (n = 255) within genetic markers with overlapped significant and suggestive associations (Table S1) with bone strength and compositional traits in White Leghorn laying hens.

| Chromosome | Region | Gene symbols | Number of SNPs | Number of genes |
| --- | --- | --- | --- | --- |
| 1 | 128422755 - 128422755 | NLGN4L | 1 | 1 |
| 1 | 173536168 - 173750242 | LOC107052041, SUPT20H, EXOSC8, ALG5, SMAD9, RFXAP, SERTM1 | 7 | 7 |
| 1 | 55553280 - 55662347 | WASHC3, DRAM1, GNPTAB, SYCP3, CHPT1, MYBPC1, LOC112533534, TRNAV-CAC, SPIC, PARP12, TBXAS1 | 3 | 11 |
| 2 | 3515191 - 3576550 | MYL3, LOC101752077, PTH1R | 2 | 3 |
| 2 | 37816681 - 37863392 | RARB, TOP2B, NGLY1, OXSM, LOC112531716 | 2 | 5 |
| 3 | 79410376 - 79578304 | SH3BGRL2, LCA5, HMGN3, PHIP, MIR6670, LOC421856, IRAK1BP1 | 5 | 7 |
| 3 | 85347154 - 85535433 | EYS, LOC112532136, LOC112532135, PHF3 | 4 | 4 |
| 3 | 9472154 - 9472154 | LOC107052015, MSH6, FBXO11, LOC107052014, LOC101749318, LOC107052024, FOXN2, PPP1R21 | 1 | 8 |
| 3 | 9671538 - 9671538 | PPP1R21, COMMD1, LOC112532216, B3GNT2, LOC421285, TMEM17, LOC101749734 | 1 | 7 |
| 4 | 51669500 - 51669500 | CENPC, STAP1, UBA6, TMPRSS11D, TMPRSS11F, YTHDC1 | 1 | 6 |
| 5 | 475797 - 475797 | DAGLA, C5H11ORF9, TMEM258, FEN1, COR8, OLFR4, OR8D4, OLFR1019L2, OLFR1019L1, TBX10, TCIRG1, NDUFS8, ALDH3B1L2, ALDH3B1L1, UNC93B1, MAX, FNTB, RAB15, GPX2, CHURC1, SPTB | 1 | 21 |
| 5 | 57086955 - 57086955 | MDGA2, LOC107053605 | 1 | 2 |
| 5 | 58459769 - 58459769 | ERO1A, PSMC6, STYX, GNPNAT1, FERMT2, DDHD1 | 1 | 6 |
| 6 | 25178685 - 25194543 | SLK, COL17A1, SFR1, CFAP43, GSTO1, ITPRIP, CFAP58 | 3 | 7 |
| 7 | 3496015 - 3528136 | ERBB4, LOC424007, LOC112532825 | 2 | 3 |
| 8 | 4316225 - 4316225 | UHMK1, C1orf226, C8H1ORF111, NOS1AP, TMEM121, LOC107053926, C8H14ORF80, CRIP1, CRIP2, MTA1 | 1 | 10 |
| 10 | 18537051 - 18683065 | IGDCC4, LOC107054244, DPP8, HACD3, VWA9, SLC24A1, DENND4A, LOC107054242, TRNAQ-CUG, RAB11A, MEGF11 | 6 | 11 |
| 10 | 21042272 - 21042272 | LOC107050231, LOC112533171 | 1 | 2 |
| 17 | 963988 - 995536 | EXFABP, LCN15, PTGDS, C8G, FBXW5, TRAF2, LOC112529985, EDF1, MAMDC4, PHPT1, LOC112529998, RABL6, AMBP, NRARP, EXD3 | 3 | 15 |
| 19 | 137076 - 517857 | LOC112530086, LOC112529957, LOC107054771, MYH7, NAA38, LOC107054772, AHDC1, DDX25, LOC107057188, ODF3L6, LOC107050024, LOC107057249, LOC107057189, BORL1, BORL4, LOC107057197, LOC112530087, LOC112530088, ODF3L15, LOC430700, TNXB, VPS37D, DNAJC30, WBSCR22, STX1A, MKS1, BCL7B, TBL2, MLXIPL, LOC107054774, ANGPTRL2, ANGPT1L, ANGPTRL1, BAZ1B, FZD9, FKBP6, TRIM50, NSUN5, POM121CL, HIP1, CCLL4, TRIM58, CCL4, CCL5, TRNAS-UGA, LPO, AGPAT1, MPO | 6 | 48 |
| 20 | 11551097 - 11551097 | LOC101748767, PMEPA1, LOC107054839, PCK1, LOC419322, LOC107054842, RBM38, LOC107054840, RAE1, SPO11, LOC112530128 | 1 | 11 |
| 20 | 11591655 - 12092729 | PMEPA1, LOC107054839, PCK1, LOC419322, LOC107054842, RBM38, LOC107054840, RAE1, SPO11, LOC112530128, BMP7, LOC107054838, LOC107054843, TFAP2C, RTFDC1, GCNT7, CSTF1, AURKA, FAM210B, LOC419333, MC3R, CBLN4, LOC419335 | 17 | 23 |
| 20 | 11828660 - 12290992 | LOC107054838, LOC107054843, TFAP2C, RTFDC1, GCNT7, CSTF1, AURKA, FAM210B, LOC419333, MC3R, CBLN4, LOC419335, DOK5, PFDN4, LOC107054836, CYP24A1, LOC107054821, BCAS1 | 15 | 18 |
| 20 | 12151445 - 12151445 | LOC419333, MC3R, CBLN4, LOC419335 | 1 | 4 |
| 22 | 2488867 - 2488867 | LOC107054965, LOC112530177, LOC107054964, ZNF703, ERLIN2, PROSC, ADGRA2, BRF2, RAB11FIP1, PRLHRL, ADRB3, EIF4EBP1, NODAL, ASH2L, STAR, LSM1, BAG4, DDHD2 | 1 | 18 |
| 22 | 746031 - 932152 | DPYSL2, BNIP3L, PPP2R2A, LOC101751589, EBF2, LOC107054950, CDCA2, KCTD9, GNRH1, DOCK5 | 4 | 10 |
| 28 | 2066107 - 2099210 | MUC16, LOC107055343, LOC107055344, LOC112530460, LOC100859850, LINGO3, C19orf35, OAZ1, AMH, SF3A2, PLEKHJ1, DOT1L, LOC112530434, AP3D1 | 3 | 14 |

# **Table S4** Known functions of genes located within 100 kb upstream or downstream of significant SNPs (*P* < 1 ×10^-5^) identified in the GWAS.

| Chr | Gene start position | Gene end position | Gene symbol | Gene description | Known functions |
| --- | --- | --- | --- | --- | --- |
| 1 | 55701730 | 55729543 | SPIC | Spi-C transcription factor | Regulates the development of bone marrow macrophages and play roles in osteoclast differentiation |
| 1 | 55597282 | 55669221 | MYBPC1 | myosin binding protein C, slow type | Primarily acts in skeletal muscles |
| 1 | 55492480 | 55507806 | DRAM1 | DNA damage regulated autophagy modulator 1 | Affects the organization and function of the Golgi apparatus |
| 1 | 55519646 | 55560185 | GNPTAB | N-acetylglucosamine-1-phosphate transferase alpha and beta subunits | Plays role in bone mineral density and content in mice |
| 1 | 55750241 | 55990122 | TBXAS1 | thromboxane A synthase 1 | May be important for bone remodeling, and the production of red blood cells in bone marrow |
| 1 | 55638160 | 55655494 | LOC112533534 | uncharacterized | - |
| 1 | 55570859 | 55591296 | CHPT1 | choline phosphotransferase 1 | May be involved in lipid metabolic pathways |
| 1 | 55672492 | 55672564 | TRNAV-CAC | transfer RNA valine (anticodon CAC) | Provides instructions for making one of several calcium channels |
| 1 | 55559464 | 55572402 | SYCP3 | synaptonemal complex protein 3 | Related to fertility traits |
| 1 | 55732612 | 55749486 | PARP12 | Poly (ADP-ribose) polymerase family member 12 | Involved in the control of protein translation and inflammation |
| 1 | 55450007 | 55465265 | WASHC3 | WASH Complex Subunit 3 | Expressed in bone marrow, predicted to be involved in actin filament polymerization and exocytosis |
| 20 | 11681928 | 11726093 | BMP7 | bone morphogenetic protein 7 | Plays role in various biological processes, including in skeletal morphogenesis |
| 20 | 11546757 | 11549468 | LOC107054839 | uncharacterized | - |
| 20 | 11629015 | 11638457 | LOC107054840 | uncharacterized | - |
| 20 | 11595229 | 11599330 | LOC107054842 | uncharacterized | - |
| 20 | 11740131 | 11745481 | LOC107054843 | uncharacterized | - |
| 20 | 11647962 | 11649570 | LOC112530128 | uncharacterized | - |
| 20 | 11591333 | 11608894 | LOC419322 | uncharacterized | - |
| 20 | 11554555 | 11562277 | PCK1 | phosphoenolpyruvate carboxykinase 1 | is essential for kidney tubular cell acid-base control, mitochondrial function, and glucose/lactate homeostasis [64]. |
| 20 | 11499475 | 11533226 | PMEPA1 | prostate transmembrane protein, androgen induced 1 | reported to be induced by testosterone or its derivatives, has been shown to have a role in tumorigenesis [65] |
| 20 | 11637658 | 11646315 | RAE1 | ribonucleic acid export 1 | is involved in mRNA export and is known to serve as a mitotic checkpoint regulator [66] |
| 20 | 11615331 | 11629002 | RBM38 | RNA binding motif protein 38 | a crucial RNA-binding protein involved in the regulation of post-transcriptional gene expression [67] |
| 20 | 11646409 | 11680108 | SPO11 | initiator of meiotic double strand breaks | Initiates meiotic recombination - a process that is essential for fertility and genetic diversity Tang el. al 2025 |
| 20 | 11685893 | 11926595 | LOC107054838 | uncharacterized | - |
| 20 | 11740131 | 11745481 | LOC107054843 | uncharacterized | - |
